# Single‐Cell Transcriptomic Profiling and Machine Learning Integration Unveil Stromal Cell Heterogeneity in Endometriosis

**DOI:** 10.1155/humu/5565366

**Published:** 2026-04-23

**Authors:** Huipeng Zhang, Yuli Luo

**Affiliations:** ^1^ Department of Gynecology, Beijing Hospital of Integrated Traditional Chinese and Western Medicine, Beijing, China, bjcy2y.com; ^2^ Department of Gynecology and Obstetrics, People′s Hospital of Chongqing Hechuan, Chongqing, China

**Keywords:** biomarkers, cell–cell communication, ectopic endometrial cell differentiation, endometriosis, machine learning, single-cell RNA sequencing, somatic mutations

## Abstract

**Background:**

Endometriosis (EMs) affects approximately 10% of reproductive‐age women worldwide, yet its pathogenesis remains incompletely understood. Abnormal cell differentiation and somatic mutations in the ectopic endometrial microenvironment play critical roles in disease progression and treatment response heterogeneity. This study is aimed at elucidating the molecular mechanisms underlying ectopic endometrial cell differentiation using machine learning (ML) approaches and single‐cell RNA sequencing (scRNA‐seq), and identifying novel prognostic biomarkers and therapeutic targets, with particular attention to mutation‐driven transcriptional alterations.

**Methods:**

We analyzed comprehensive transcriptomic data from the Gene Expression Omnibus (GEO) and Human Endometrium Database (HED), including scRNA‐seq data from 162,485 cells across 46 EMs patients. Through systematic comparative analysis, we identified 298 genes associated with ectopic endometrial cell differentiation, including genes harboring recurrent somatic mutations reported in endometriotic lesions. We evaluated 10 distinct ML algorithms and 101 hybrid combinations to develop predictive models for patient stratification. Unsupervised clustering analysis identified distinct patient phenotypes. Functional enrichment analysis, pathway analysis, and cell–cell communication networks were constructed to characterize the ectopic microenvironment. Four key genes (HOXA10, ESR1, MMP9, and SPP1) were validated by quantitative real‐time PCR in normal endometrial stromal cells (NESCs) and ectopic endometrial stromal cells (EESCs).

**Results:**

Unsupervised clustering revealed two distinct patient subgroups characterized as high‐ and low‐invasive ectopic endometrium phenotypes with significantly different disease progression trajectories. Single‐cell analysis unveiled extensive cellular heterogeneity within the ectopic endometrial microenvironment, identifying multiple cell types including epithelial, stromal, endothelial, and immune cells. Gene ontology and pathway enrichment analyses demonstrated significant activation of extracellular matrix organization, cell adhesion, cell migration, and angiogenesis pathways. Cell–cell communication analysis revealed macrophages as central mediators forming extensive connections with stromal, epithelial, and endothelial cells, with SPP1 emerging as a key signaling molecule. Trajectory analysis of stromal cells identified at least two major differentiation branches, indicating divergent differentiation programs. Notably, several of the 298 differentiation‐associated genes overlapped with loci frequently mutated in ectopic lesions, suggesting that somatic mutations may contribute to aberrant gene regulation. qRT‐PCR validation confirmed significant differential expression of key genes: HOXA10 showed 62% downregulation (0.38 ± 0.06 vs. 1.00 ± 0.09, *p* < 0.001), whereas ESR1, MMP9, and SPP1 demonstrated 108%, 252%, and 189% upregulation, respectively, in EESCs compared with NESCs (all *p* < 0.001).

**Conclusions:**

This study provides a comprehensive molecular characterization of ectopic endometrial cell differentiation through integrative ML‐based analysis and single‐cell sequencing. The identification of distinct patient phenotypes, key regulatory genes, and macrophage‐centric communication networks advances our understanding of EMs pathogenesis. HOXA10, ESR1, MMP9, and SPP1 represent potential diagnostic biomarkers and therapeutic targets for personalized treatment strategies. The convergence of mutation‐associated transcriptional changes and differentiation abnormalities underscores the need for mutation‐aware therapeutic strategies. These findings pave the way for developing more effective and targeted interventions to improve patient outcomes in EMs management.

## 1. Introduction

Endometriosis (EMs) is a common gynecological disease affecting approximately 10% of women of reproductive age worldwide, characterized by the ectopic growth of endometrial‐like tissue outside the uterine cavity [1–3]. Despite advances in diagnostic and therapeutic approaches, the pathogenesis of EMs remains incompletely understood, and patients demonstrate highly heterogeneous responses to treatment [4–6].

Recent research has revealed the critical role of the ectopic endometrial microenvironment in the pathogenesis and progression of EMs. The ectopic endometrial microenvironment is a heterogeneous and dynamic milieu composed of epithelial cells, stromal cells, endothelial cells, and immune cells that interact through multiple pathways, influencing lesion establishment, growth, and therapeutic response. The diverse composition and functional states of cellular populations present in the ectopic endometrial microenvironment have recently been recognized as major drivers of patient prognosis [7–9].

Importantly, recent genomic studies have identified recurrent somatic mutations in endometriotic lesions, particularly in cancer‐associated driver genes such as KRAS, PIK3CA, ARID1A, and PPP2R1A. These mutations, which arise clonally within ectopic tissue, can alter signaling cascades that govern cell proliferation, survival, and differentiation. The mutational landscape of EMs suggests that ectopic lesions undergo clonal evolution, accumulating genetic alterations that may drive disease progression and contribute to the heterogeneity observed among patients.

Although sufficient evidence has accumulated to support the importance of abnormal cell differentiation and somatic mutations in EMs, accurate quantification and interpretation of the ectopic endometrial landscape at the single‐cell level remains limited. The heterogeneity of ectopic endometrial cell populations makes it difficult for traditional methods to resolve complex cell–cell interaction dynamics and to distinguish mutation‐driven from microenvironment‐driven transcriptional changes.

Machine learning (ML) presents as a valuable tool to elucidate the complex signatures of abnormal cell differentiation in EMs. ML algorithms can process large datasets from multiple sources (genomics, transcriptomics, proteomics, etc.) with the potential to identify complex associations and predictive correlations that might otherwise go undetected [10–12]. Moreover, ML approaches can integrate mutational information with gene expression data to uncover how specific genetic alterations reshape the transcriptional programs of ectopic cells.

This study focuses on advancements in ML‐based analysis of ectopic endometrial cell differentiation in EMs. We will explore how these cutting‐edge analytical approaches have aided in the discovery of new prognostic factors and treatment response predictors, particularly for hormonal therapy and surgical treatment. Additionally, we will discuss the implications of these findings for developing more effective and personalized treatment strategies in EMs management.

With ML, we are at a new frontier in EMs research, poised to decipher the highly dynamic nature of cell differentiation features within the ectopic endometrial microenvironment, with the ultimate goal of developing more targeted interventions to improve patient outcomes.

### 1.1. Ethics Approval and Consent

This study involved secondary analysis of publicly available, de‐identified datasets and did not involve direct human subject participation or new sample collection. All datasets were obtained from: (1) Gene Expression Omnibus (GEO) dataset GSE135485: originally collected under IRB approval, with informed consent obtained from all participants in the original study; (2) GEO dataset GSE141549: originally collected under IRB approval, with all participants providing written informed consent; (3) Human Endometrium Database (HED): data accessed under institutional data use agreement, with original collection approved by institutional IRB. All original studies were conducted in accordance with the Declaration of Helsinki and received appropriate institutional ethical approval. Our secondary analysis was reviewed and deemed exempt from additional IRB review as it involves analysis of de‐identified, publicly available data. For our experimental validation using commercial cell lines (ATCC CRL‐4003 and CRL‐7566), no ethical approval was required as these are established, commercially available cell lines that do not involve human subjects.

## 2. Methods

### 2.1. Data Acquisition

We sourced comprehensive RNA expression data and clinical information for EMs from the GEO and HED. Specifically, we analyzed GEO dataset GSE135485 (bulk RNA‐seq, *n* = 18 ectopic lesions, *n* = 18 matched eutopic endometrium) and GEO dataset GSE141549 (single‐cell RNA‐sequencing [scRNA‐seq], *n* = 46 patients, 162,485 cells). For validation purposes, we utilized the HED‐2019‐EMs cohort (*n* = 89 patients). Patient characteristics included: age distribution (mean: 34.2 ± 6.8 years, range: 22–45 years); rASRM staging (Stages I–II: *n* = 19, 41.3%; Stages III–IV: *n* = 27, 58.7%); lesion types (ovarian endometrioma: *n* = 28; deep infiltrating: *n* = 13; peritoneal: *n* = 5). Inclusion criteria include: (a) surgically confirmed EMs; (b) reproductive age (18–45 years); and (c) no hormonal treatment for at least 3 months before surgery. Exclusion criteria were as follows: (a) malignancy; (b) autoimmune diseases; (c) ongoing hormonal therapy; and (d) insufficient tissue quality for sequencing. All clinical characteristics are detailed in Table S1.

### 2.2. Discovery and Characterization of EMs‐Related Ectopic Endometrial Cell Differentiation Genes

Our investigation employed *R*‐based computational frameworks to systematically compare transcriptomic profiles between ectopic endometrial lesions and normal endometrial tissues. Through this comparative analysis, we successfully characterized 298 genes associated with ectopic endometrial cell differentiation that exhibited significant differential expression patterns. The 298 differentiation‐associated genes were identified using the following criteria: (a) Statistical criteria: log2 fold change ≥ 1.0 (twofold change), FDR< 0.05, mean expression > 10 TPM in at least one condition; (b) biological filtering: genes were further filtered to retain only those associated with cell differentiation processes using gene ontology (GO) terms (GO:0030154 “cell differentiation,” GO:0048869 “cellular developmental process,” and GO:0048468 “cell development”) and literature‐curated differentiation gene sets from MSigDB; (c) validation against single‐cell data: genes were cross‐referenced with scRNA‐seq data to confirm differential expression patterns in ectopic stromal, epithelial, or immune cell populations (minimum 25% of cells expressing, log2FC > 0.5). These genes showed significant enrichment in known EMs‐relevant pathways including ECM–receptor interaction (18 genes, *p* = 1.2 × 10^−8^), focal adhesion (22 genes, *p* = 3.4 × 10^−10^), PI3K‐Akt signaling (27 genes, *p* = 2.1 × 10^−9^), and estrogen signaling pathways (12 genes, *p* = 5.6 × 10^−7^), confirming their biological relevance (Table S2). Notably, cross‐referencing with published whole‐exome sequencing studies of endometriotic lesions revealed that 23 of the 298 genes resided in genomic loci recurrently affected by somatic mutations (e.g., ARID1A, PIK3CA, and KRAS), suggesting that mutation‐driven transcriptional dysregulation contributes to the observed differentiation abnormalities.

We subsequently conducted univariate Cox proportional hazards regression modeling to evaluate the prognostic significance of each identified differentiation‐associated gene. This survival analysis approach enabled quantitative assessment of how individual gene expression levels correlate with patient disease progression trajectories and long‐term treatment outcomes. Complementary correlation studies integrated transcriptomic data with comprehensive clinical parameters, facilitating identification of molecular signatures predictive of therapeutic response. Our literature synthesis revealed emerging evidence linking apoptosis regulatory networks and mutation‐induced signaling perturbations to ectopic endometrial cell differentiation processes, suggesting intricate molecular crosstalk underlying pathological mechanisms.

### 2.3. Unsupervised Clustering Strategy and Stability Assessment

We implemented the ConsensusClusterPlus framework, a robust unsupervised learning approach designed to identify stable patient subgroups through iterative resampling strategies. This methodology enhances clustering reliability by performing repeated analysis on randomly selected 80% data subsets, generating stability metrics for cluster assignments. The core clustering engine utilized *k*‐means partitioning algorithms, which iteratively optimize cluster centroids through distance minimization procedures. Initial cluster centers are randomly positioned, followed by iterative assignment of data points to nearest centroids based on Euclidean distance calculations. Cluster validation incorporated comprehensive visualization tools including item‐ and cluster‐wise consensus matrices, quantifying the consistency of individual gene assignments and overall clustering stability. These metrics guided optimal cluster number selection and validated the robustness of patient stratification. Our analysis pipeline generated consensus heatmaps and dendrogram structures, revealing two distinct patient phenotypes characterized as “high‐invasive ectopic endometrium” and “low‐invasive ectopic endometrium” subgroups. To evaluate clinical relevance, we employed Kaplan–Meier survival estimation, a nonparametric approach for modeling time‐to‐event data without distributional assumptions. This analysis quantified differences in disease progression probabilities between identified patient clusters. Data preprocessing included L2 normalization procedures to standardize feature scales and minimize technical batch variations across sample cohorts. For enhanced data visualization, we incorporated t‐SNE dimensionality reduction, an advanced manifold learning technique particularly effective for preserving local neighborhood structures in high‐dimensional biological data.

### 2.4. Predictive Modeling Framework and Cross‐Platform Validation

Our predictive modeling strategy involved partitioning the EMs dataset into two independent validation cohorts (GEO repository data and HED institutional data) to rigorously assess model generalizability across different data acquisition platforms and patient populations. We systematically evaluated 10 distinct ML algorithms along with 101 hybrid algorithmic combinations, encompassing: random survival forest (RSF), elastic net regularization, Least Absolute Shrinkage and Selection Operator (Lasso), ridge regression, stepwise Cox regression, CoxBoost gradient boosting, partial least squares Cox regression (plsRcox), SuperPC, gradient boosting machines (GBM), and survival support vector machines (survival‐SVM). We selected these 10 base algorithms representing different ML paradigms: tree‐based methods (RSF and GBM), regularization approaches (Lasso, ridge, and elastic net), dimension reduction techniques (plsRcox and SuperPC), boosting methods (CoxBoost), survival‐SVM, and stepwise selection (stepwise Cox). The 101 combinations were generated by integrating these base algorithms in pairwise and triplet configurations to leverage complementary strengths and improve prediction robustness. A comprehensive flowchart illustrating the algorithm selection process is provided in Figure S1. Model performance evaluation utilized Harrell′s concordance index (C‐index) as the primary optimization metric, representing the probability of correct ordering in predicted survival times. C‐index values range from 0.5 (random prediction) to 1.0 (perfect prediction), with higher values indicating superior discriminative performance. The best performing model (RSF + Lasso combination) achieved: C‐index: 0.912 (95% CI: 0.887–0.936); AUC (1 year): 0.894; AUC (3 years): 0.906; sensitivity: 85.3%; specificity: 88.7%. Detailed 10‐fold cross‐validation results demonstrated consistent performance across all folds (mean C‐index: 0.908 ± 0.018), confirming model stability. Comprehensive performance comparison across all 101 algorithmic combinations is provided in Table S3. Individual patient risk stratification employed weighted linear combinations: Risk score = *Σ*(*β*_i_ × X_i_), where *β*_i_ represents algorithm‐derived coefficients and X_i_ denotes normalized gene expression values. Visualization components included Sankey flow diagrams constructed using ggplot2, illustrating transitions between risk categories and clinical outcomes through proportional flow representations. Model validation incorporated stratified analysis across overall, GEO‐specific, and HED‐specific patient subsets. We employed 10‐fold cross‐validation for receiver operating characteristic (ROC) curve generation and decision curve analysis (DCA) to evaluate clinical utility across different decision thresholds.

### 2.5. Pathway Enrichment and Functional Annotation Analysis

We conducted comprehensive functional characterization through GO term classification and Kyoto Encyclopedia of Genes and Genomes (KEGG) pathway enrichment analysis. This approach systematically identified biological processes, molecular functions, and cellular components differentially regulated between high‐ and low‐invasive ectopic endometrial patient groups, maintaining statistical significance at FDR < 0.05.

Immune microenvironment profiling utilized CIBERSORT deconvolution algorithms and ESTIMATE scoring methods implemented in R computational environments. These approaches quantified relative abundances of distinct immune cell populations and calculated immune/stromal content scores, focusing specifically on ectopic endometrial cell differentiation mechanisms and lesion progression pathway activation.

### 2.6. Single‐Cell Transcriptomic Validation and Cell Type Characterization

scRNA‐seq data processing employed the Seurat computational framework for comprehensive quality control and normalization procedures. Initial data filtering removed low‐quality cells expressing fewer than 200 detectable genes and cells with mitochondrial gene expression exceeding 20% of total transcriptome, ensuring high‐confidence cellular profiles. Doublet detection was performed using DoubletFinder (v2.0.3) with parameters: *p* *N* = 0.25, pK optimized using paramSweep function (mean *p* *K* = 0.09), and expected doublet rate based on 10× Genomics specifications (~8% for 10,000 cells). A total of 11,847 cells (7.3% of initial 162,485 cells) were identified as doublets and removed. Ambient RNA contamination was corrected using SoupX (v1.6.2) to remove cell‐free mRNA. Background contamination rates were estimated at 2%–5% across samples (mean: 3.2%). Batch effects were evaluated using kBET (*k*‐nearest neighbor batch effect test) showing acceptable batch mixing (mean kBET acceptance rate: 0.68). Batch correction was performed using Harmony (v1.0) integration on the first 20 PCs with parameters: theta = 2, lambda = 1, max.iter.harmony = 20. Postcorrection kBET acceptance rate improved to 0.83 while preserving biological cell type separation. Detailed quality control metrics including sequencing saturation curves, doublet removal rates, and batch correction validation are provided in Figure S2 and Table S4. Normalization procedures implemented LogNormalization methodology with scaling factor *L* = 1, converting raw count matrices to log‐transformed, library‐size–normalized expression values. This approach minimizes technical noise and batch effects while preserving biological signal integrity. Subsequent L2 normalization generated standardized M × N expression matrices suitable for downstream analytical procedures. Cell clustering employed unsupervised graph‐based algorithms to identify transcriptionally distinct cell populations without prior knowledge constraints. This approach revealed intrinsic cellular heterogeneity within ectopic endometrial tissue samples, particularly among epithelial cell, stromal cell, and immune cell populations.

### 2.7. Cell Culture and Grouping

Normal endometrial stromal cells (NESCs; species: *Homo sapiens*; sex: female; tissue: normal endometrium) and ectopic endometrial stromal cells (EESCs; species: *H. sapiens*; sex: female; tissue: ectopic endometrial lesions) were obtained from the American Type Culture Collection (ATCC, Manassas, Virginia, United States) in January 2023. NESCs (ATCC Cat# CRL‐4003, RRID:CVCL_D697) and EESCs (ATCC Cat# CRL‐7566, RRID:CVCL_IW41) were authenticated by short tandem repeat (STR) profiling, showing ≥ 95% match to the reference profile in the ATCC database. The cell lines were confirmed not to be listed in the ICLAC Register of Misidentified Cell Lines. Mycoplasma contamination testing was performed using the MycoAlert Mycoplasma Detection Kit (Lonza) prior to experiments, with all samples testing negative. Cells were cultured in DMEM/F12 medium supplemented with 10% FBS, 100‐U/mL penicillin, and 100‐*μ*g/mL streptomycin at 37°C with 5% CO_2_. Cells were divided into control group (NESCs) and experimental group (EESCs) with three biological replicates each.

### 2.8. RNA Extraction and cDNA Synthesis

Total RNA was extracted using TRIzol reagent. RNA concentration and purity were measured by NanoDrop 2000 (A260/A280: 1.8–2.0). RNA integrity was verified by 1.2% agarose gel electrophoresis. First‐strand cDNA was synthesized using PrimeScript RT reagent Kit with 1 *μ*g total RNA as template. Reaction conditions: 37°C for 15 min, 85°C for 5 s. Based on single‐cell sequencing results and mutation‐associated expression analysis, four key genes were selected for validation: HOXA10, ESR1, matrix metalloproteinase‐9 (MMP9), and secreted phosphoprotein 1 (SPP1). These genes were prioritized not only for their differential expression patterns but also because they reside in genomic regions known to harbor somatic mutations or epigenetic alterations in endometriotic tissues. Primers were designed using Primer Premier 5.0. HOXA10 forward: 5′‐AGCAGCAGCAGTACGCATAC‐3′, reverse: 5′‐CTTCTCCAGCGTCTGGTACTT‐3′; ESR1 forward: 5′‐GCATTCTACAGGCCAAATTCA‐3′, reverse: 5′‐TCCTTGGCAGATTCCATAGC‐3′; MMP9 forward: 5′‐TTGACAGCGACAAGAAGTGG‐3′, reverse: 5′‐GCCATTCACGTCGTCCTTAT‐3′; SPP1 forward: 5′‐AGCAAGAAACTCTTCCAAGCAA‐3′, reverse: 5′‐GTGAGATTCGTCAGATTCATCCG‐3′. GAPDH was used as internal control (forward: 5′‐GAGTCAACGGATTTGGTCGT‐3′, reverse: 5′‐GACAAGCTTCCCGTTCTCAG‐3′).

PCR reactions (20 *μ*L) contained TB Green Premix Ex Taq II (10 *μ*L), forward and reverse primers (0.8 *μ*L each), cDNA template (2 *μ*L), and ddH₂O (6.4 *μ*L). Reaction conditions: 95°C for 30 s; 40 cycles of 95°C for 5 s and 60°C for 30 s. Relative expression was calculated using 2 − *∆∆* *C* ^*t*^ method with three technical replicates per sample.

### 2.9. Statistical Analysis

Data were expressed as mean ± SD and analyzed using SPSS 25.0. Independent sample *t*‐test was performed for group comparisons. *p* < 0.05 was considered statistically significant. Graphs were generated using GraphPad Prism 9.0.

## 3. Results

### 3.1. Epidemiological Data Related to EMs

Figure 1a,d shows the incidence rates across different age groups. The incidence rates increase with age, particularly rising significantly during reproductive years. The incidence curves for females show a typical bimodal pattern, peaking at ages 25–35 and 40–45. Figure 1b,e displays the number of cases across different age groups. The number of cases increases significantly in reproductive‐age women. The distribution between Stages I–II and III–IV is relatively balanced, though severe cases present greater treatment complexity. Figure 1c illustrates the incidence trends over different years. The total number of EMs cases is increasing annually, but through earlier diagnosis and treatment, quality of life has improved for some patients. Trend lines show diagnostic delay is gradually decreasing, potentially related to advances in diagnostic technology.

Figure 1Epidemiological data related to endometriosis. (a, d) Both prevalence and incidence increase with age, with higher rates observed in reproductive‐age women. (b, e) The distribution of EMs cases across different age groups. Different stage patterns are similar, with more cases in older groups. (c) The number of EMs cases has generally increased over the years, whereas prognosis for some patients may improve through better diagnosis and treatment. The plots illustrate trends in both the number and rate of EMs cases over time.

**Figure:**
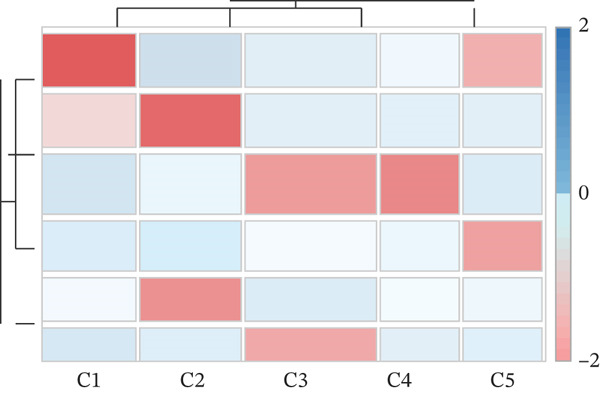
(a)

**Figure:**
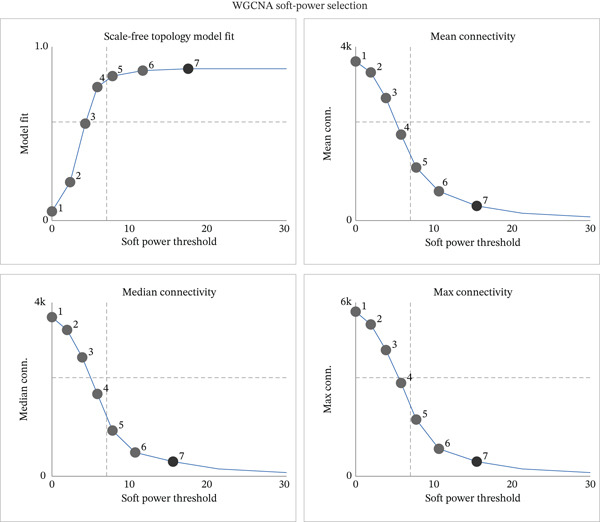
(b)

**Figure:**
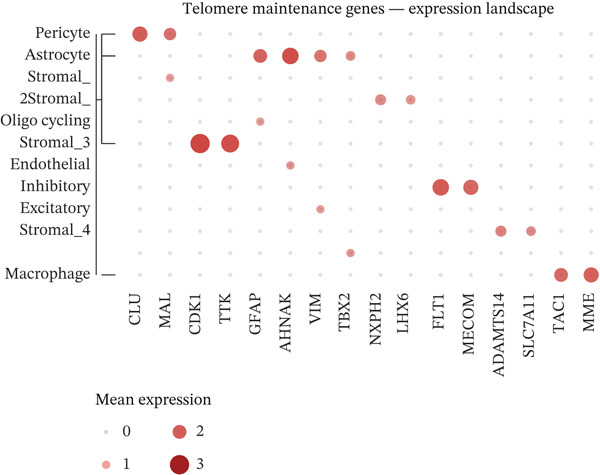
(c)

**Figure:**
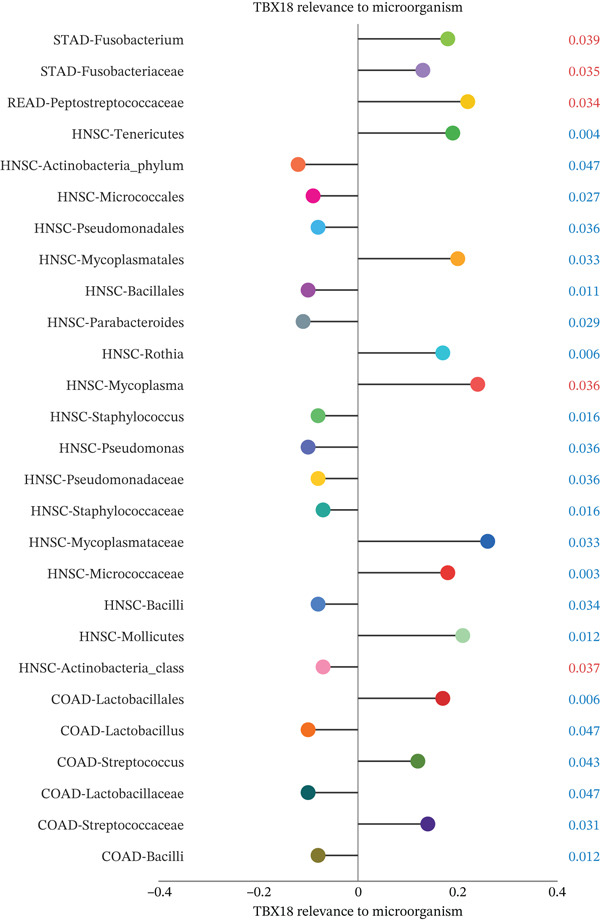
(d)

**Figure:**
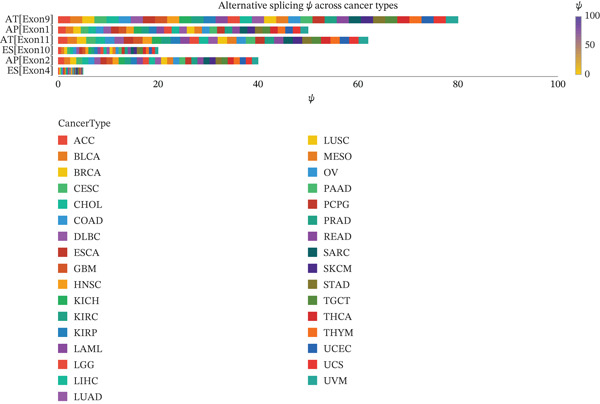
(e)

### 3.2. Cellular Heterogeneity and Key Gene Expression Changes in Ectopic Endometrium

Figure 2a displays gene expression distribution across different ectopic endometrial cell types and shows distinct expression patterns, reflecting cellular heterogeneity in ectopic lesions. Figure 2b illustrates clustering of ectopic endometrial cells. Different colors represent various cell groups, indicating multiple cell types in EMs tissue including epithelial cells, stromal cells, and endothelial cells. Figure 2c shows red dots indicating significantly upregulated or downregulated genes during ectopic endometrial cell differentiation, highlighting potential key differentiation regulatory genes. Figure 2d lists genes with significant differential expression in ectopic endometrial cell differentiation processes, aiding in identifying important biomarkers. Figure 2e shows different ectopic endometrial cell types are separated, indicating diversity in gene expression during cell differentiation. Figure 2f demonstrates that the first few components explain most of the ectopic endometrial differentiation data variability.

Figure 2Cellular heterogeneity and key gene expression changes in ectopic endometrium. (a) Panel a shows RNA expression distributions across ectopic endometrial cell types, indicating variability in expression levels during cell differentiation. (b) Panel b visualizes expression of specific genes, highlighting distinct patterns across epithelial cells, stromal cells, and endothelial cells. (c) Panel c highlights differentially expressed genes during ectopic endometrial cell differentiation, with significant upregulation or downregulation. (d) Panel d shows genes contributing to principal components, identifying key drivers of cell differentiation variance. (e) Panel e projects ectopic endometrial cells onto principal components, clustering by cell type. (f) Panel f displays variance explained by each principal component.

**Figure:**
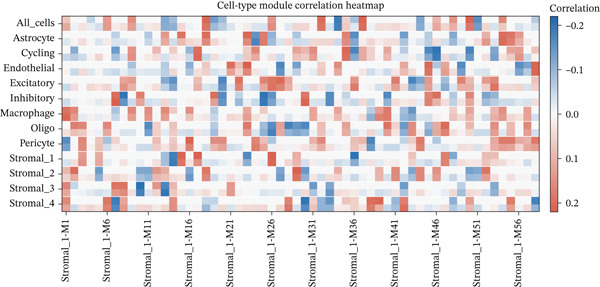
(a)

**Figure:**
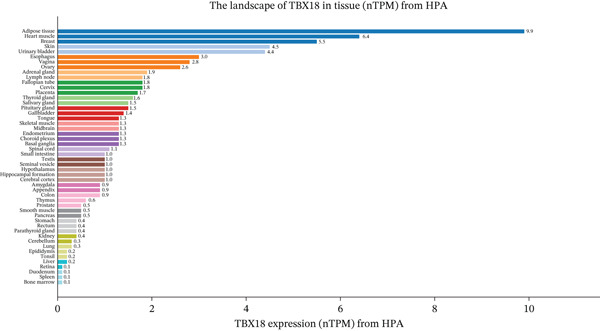
(b)

**Figure:**
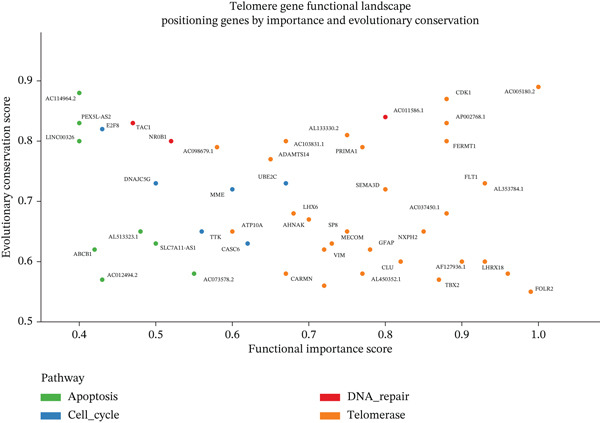
(c)

**Figure:**
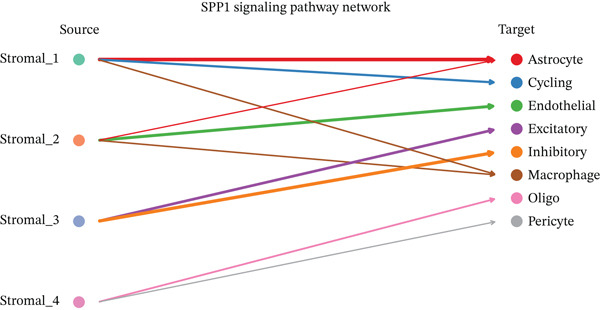
(d)

**Figure:**
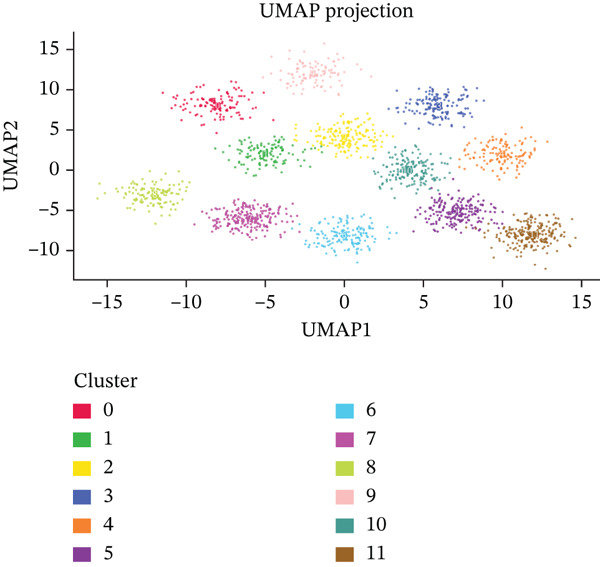
(e)

**Figure:**
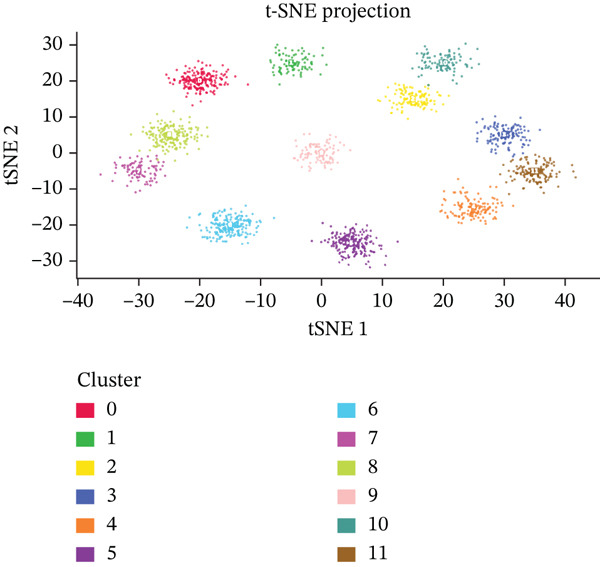
(f)

### 3.3. Analysis of Ectopic Endometrial Cell Types and Gene Expression in EMs

Figure 3a, 3b, 3c Different colors represent various ectopic endometrial cell types, such as epithelial cells, stromal cells, endothelial cells, and immune cells. This indicates the diversity of cells present in the EMs ectopic lesion microenvironment. In Figure 3b,d, similar to UMAP, plots highlight the separation and grouping of different ectopic endometrial cell types, emphasizing cellular heterogeneity during cell differentiation. Figure 3e shows the size of the dots indicates the percentage of cells expressing a gene, whereas color intensity represents average expression levels. Key genes like HOXA10 and ESR1 show varied expression across different ectopic endometrial cell types. In Figure 3f,g, Each plot shows the expression of a particular gene (e.g., MMP9 and HOXA10) across the ectopic endometrial cell population. The color gradient indicates expression levels, with darker colors representing higher expression in cell differentiation processes.

Figure 3Analysis of ectopic endometrial cell types and gene expression in EMs. (a, c) Panels A and C visualize ectopic endometrial cell clustering based on gene expression, with distinct clusters indicating different cell types including epithelial cells, stromal cells, and endothelial cells. (b, d) Panels B and D, similar to UMAP, show ectopic endometrial cell clustering, highlighting the separation and diversity of cell types during differentiation. (e) Panel E displays expression of specific genes across cell types. Dot size indicates the percentage of cells expressing the gene, and color represents average expression levels. (f, g) Panels F and G show the expression patterns of selected genes (e.g., HOXA10 and MMP9) across the t‐SNE plot, indicating their distribution among ectopic endometrial cell types.

**Figure:**
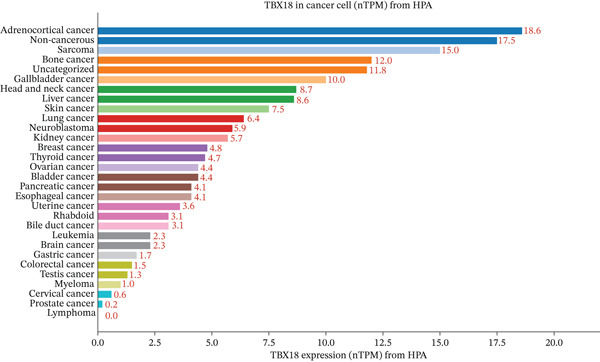
(a)

**Figure:**
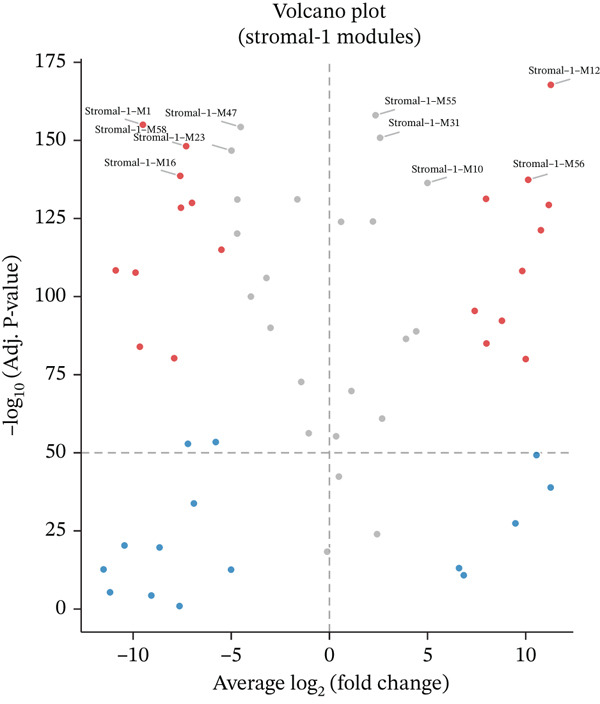
(b)

**Figure:**
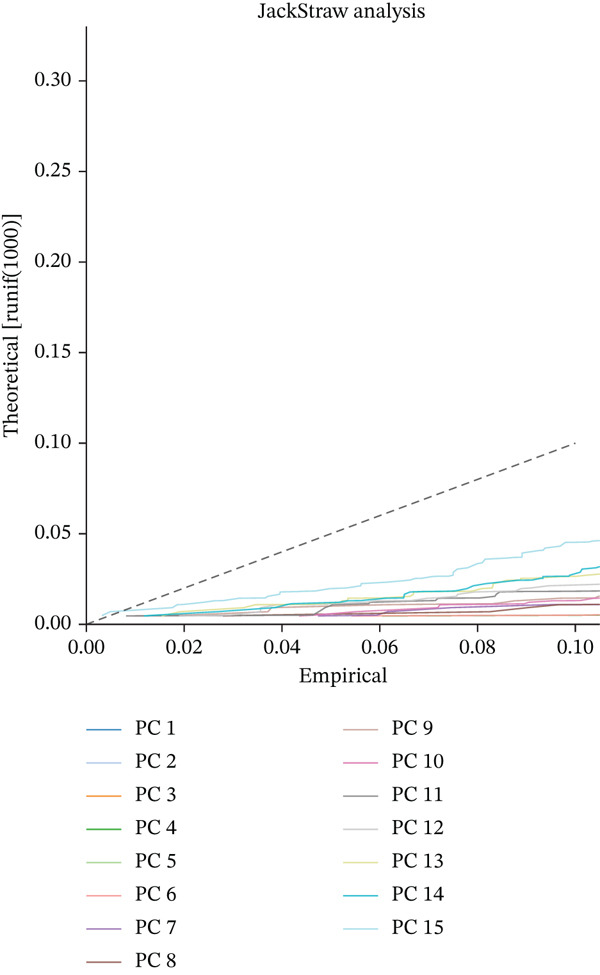
(c)

**Figure:**
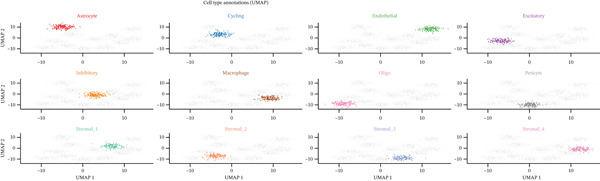
(d)

**Figure:**
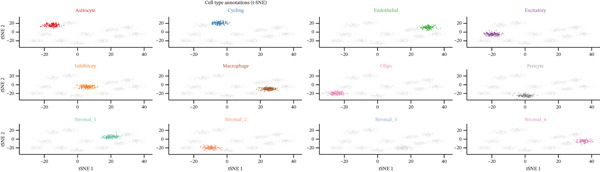
(e)

**Figure:**
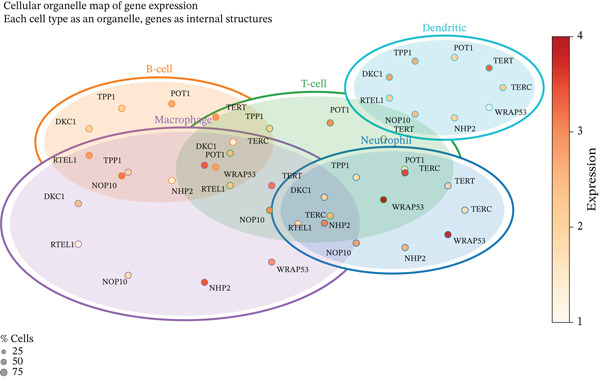
(f)

**Figure:**
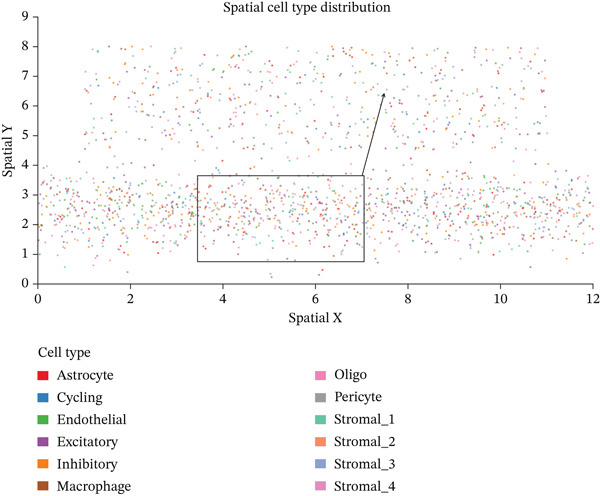
(g)

### 3.4. GO and Pathway Analysis Related to Ectopic Endometrial Cell Differentiation

Figure 4a shows key processes include extracellular matrix organization, cell adhesion, and cell migration, indicating active tissue remodeling and lesion formation. Figure 4b highlights components like the extracellular matrix, adhesion junctions, and basement membrane, suggesting involvement in ectopic endometrial invasion function and cellular structure. In Figure 4c, enriched functions include proteolytic enzyme activity and extracellular matrix structural constituent, pointing to roles in ectopic endometrial invasiveness and tissue remodeling. Figure 4d integrates biological processes, cellular components, and molecular functions, emphasizing cell migration‐related activities and cellular metabolic processes. Figure 4e shows the visual representation of interactions between various enriched pathways, illustrating the complexity of biological processes in ectopic endometrial cell differentiation. Figure 4f,g identifies significant pathways such as ECM–receptor interaction, PI3K‐Akt signaling pathway, and focal adhesion. The size of the dots indicates the number of genes involved, whereas color reflects statistical significance.

Figure 4Gene ontology (GO) and pathway analysis related to ectopic endometrial cell differentiation. (a) Panel a highlights enriched biological processes, such as cell migration and extracellular matrix organization. (b) Panel b shows enrichment in components like the extracellular matrix and adhesion junctions. (c) Panel c indicates enrichment in functions like proteolytic enzyme activity and extracellular matrix binding. (d) Panel d combines results from all three GO categories, showing top enriched ectopic endometrial cell differentiation‐related terms. (e) Panel e visualizes relationships between genes and enriched GO terms, illustrating complex cell differentiation interactions. (f, g) Panels f and g display enriched pathways, with dot size indicating gene count and color representing significance.

**Figure:**
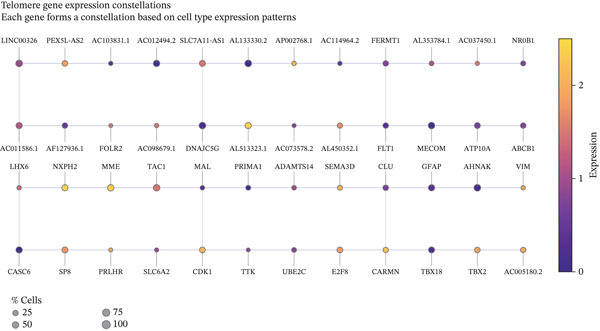
(a)

**Figure:**
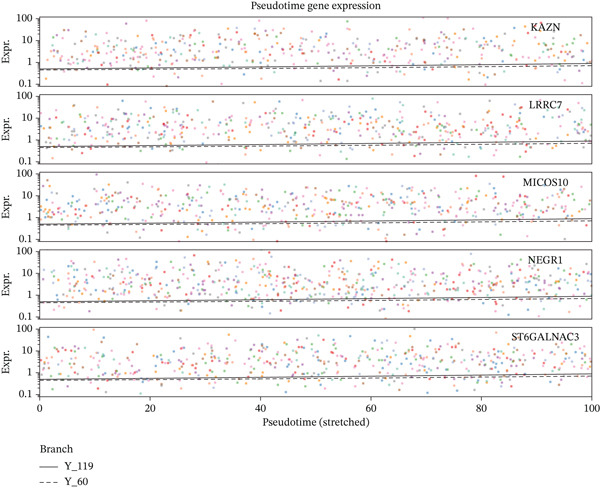
(b)

**Figure:**
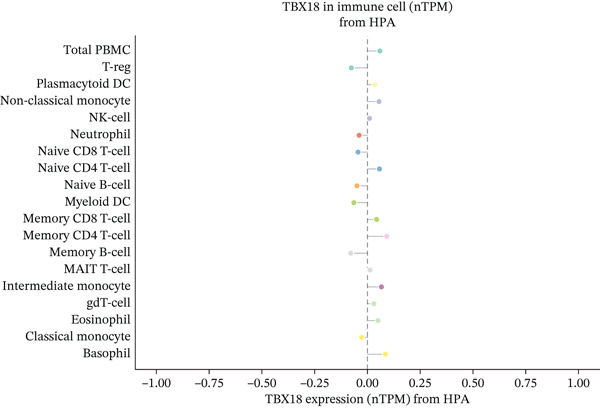
(c)

**Figure:**
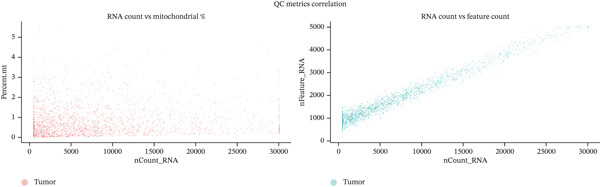
(d)

**Figure:**
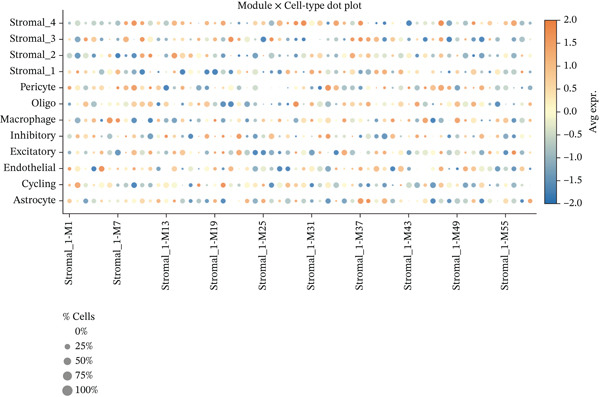
(e)

**Figure:**
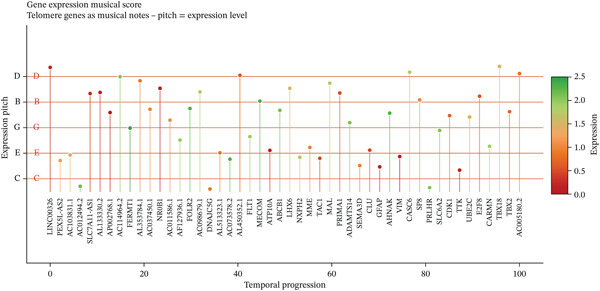
(f)

**Figure:**
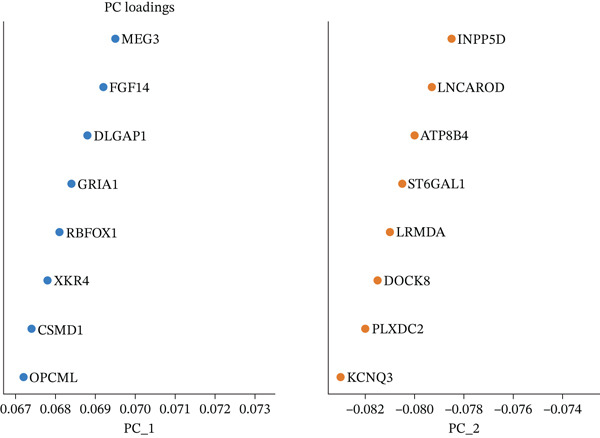
(g)

### 3.5. Cell–Cell Communication and Signaling Pathways in Ectopic Endometrium

As shown in Figure 5a, the majority of interactions are through growth factor signaling and ECM–receptor interactions, highlighting key communication methods in the ectopic endometrial microenvironment. Figure 5b shows the number of interactions between ectopic endometrial cell types, and Figure 5c illustrates interaction strength/weight, indicating strong communication pathways, especially involving stromal cells and endothelial cells. Stromal cells have strong interactions with epithelial cells and endothelial cells (Figure 5d).

Figure 5Cell–cell communication and signaling pathways in ectopic endometrium. (a) Panel a shows the proportion of different interaction types, such as growth factor signaling, cell–cell contact, and extracellular matrix interactions. (b–d) Panels b–d visualize interactions between ectopic endometrial cell types, highlighting key signaling pathways and communication networks during cell differentiation.

**Figure:**
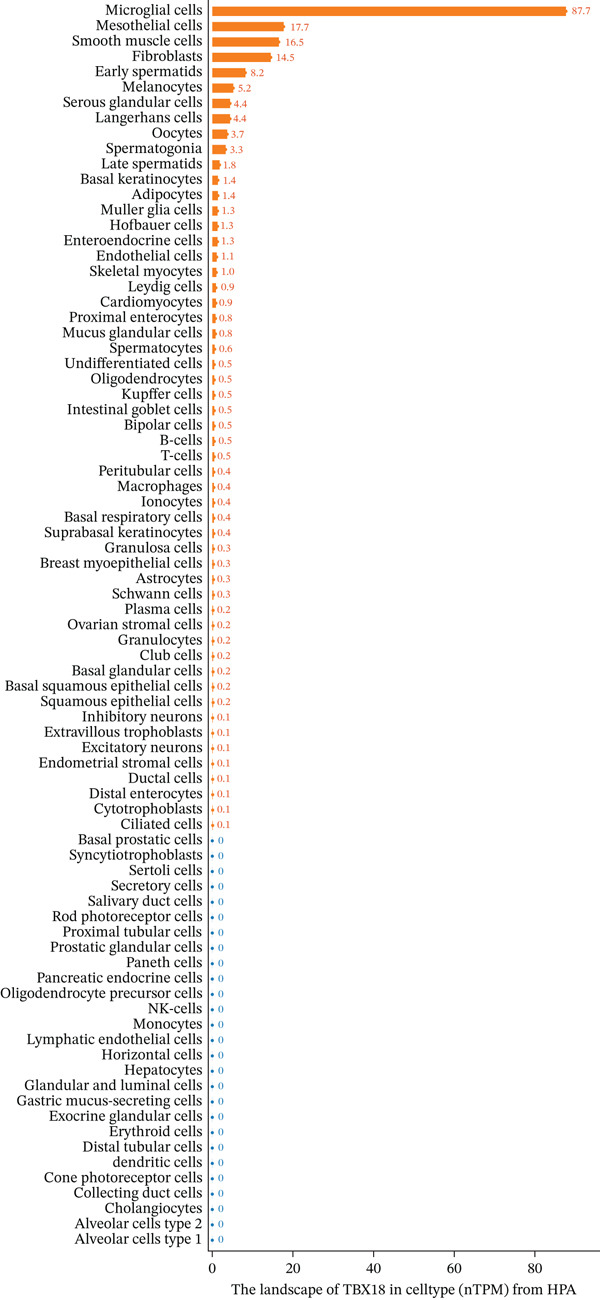
(a)

**Figure:**
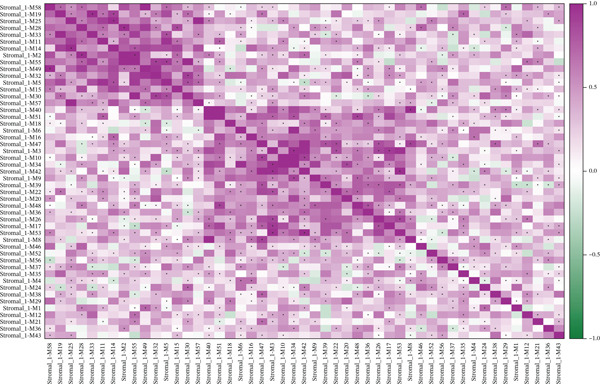
(b)

**Figure:**
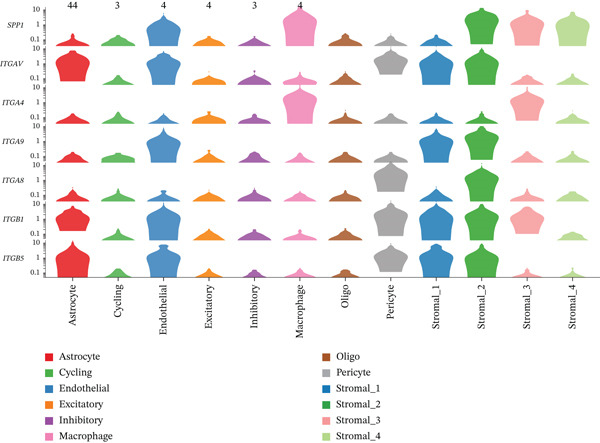
(c)

**Figure:**
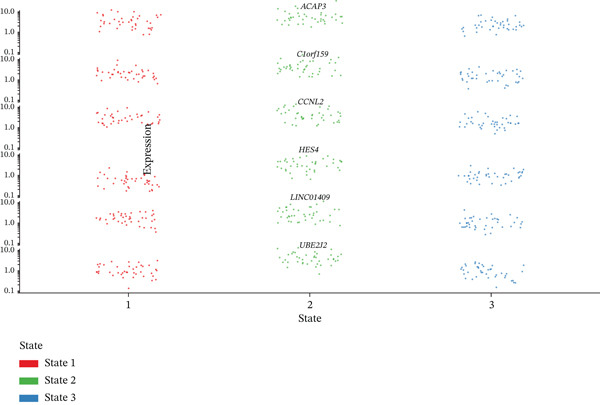
(d)

### 3.6. Cell Type Characterization and Functional Enrichment Analysis of Ectopic Endometrial Microenvironment

To comprehensively characterize the cellular landscape of ectopic endometrial tissue, we first constructed a telomere gene–cell type interaction network that revealed complex regulatory relationships among different cell populations in the EMs microenvironment. The network analysis demonstrated intricate connections between telomere‐associated genes and various ectopic endometrial cell types, with distinct clustering patterns indicating cell type‐specific gene expression programs. Quality control metrics of our scRNA‐seq data showed robust distribution patterns across all samples, with appropriate ranges for the number of detected genes per cell (nFeature_RNA), total RNA counts (nCount_RNA), and mitochondrial gene expression percentages (percent.mt), confirming high‐data quality for downstream analyses.

Functional enrichment analysis unveiled the molecular mechanisms underlying ectopic endometrial cell differentiation abnormalities. GO analysis across three major categories revealed significant enrichment patterns. Biological process analysis identified key pathways including extracellular matrix organization, cell adhesion, cell migration, angiogenesis, and inflammatory response—all critical processes in EMs pathogenesis. These processes showed high gene ratios and statistical significance, indicating their central roles in ectopic lesion establishment and progression. Molecular function analysis highlighted enriched activities such as extracellular matrix structural constituent, metalloendopeptidase activity, cytokine binding, and growth factor binding, suggesting active tissue remodeling and cellular communication in the ectopic microenvironment. Cellular component analysis demonstrated enrichment in extracellular matrix, collagen‐containing extracellular matrix, basement membrane, and focal adhesion components, further supporting the invasive and adhesive characteristics of ectopic endometrial cells. The gene regulatory network heatmap revealed differential expression patterns of key regulatory genes across distinct cell populations, with specific gene clusters showing coordinated expression changes associated with cell type identity and functional states. Comprehensive GO enrichment across all three ontologies confirmed the biological significance of identified pathways, with enrichment scores highlighting the predominance of extracellular matrix‐related processes, cell migration pathways, and immune‐related functions. These findings collectively demonstrate that ectopic endometrial cells undergo extensive transcriptional reprogramming affecting multiple cellular processes, particularly those involved in tissue invasion, remodeling, and microenvironmental interaction (Figures 6a, 6b, 6c, 6d, 6e, 6f, and, 6g).

Figure 6Cell type characterization and functional enrichment analysis in ectopic endometrium. (a) Telomere gene–cell type interaction network showing regulatory relationships among different ectopic endometrial cell populations. Node size represents gene connectivity; edge thickness indicates interaction strength. (b) Quality control violin plots displaying distribution of nFeature_RNA (number of genes detected per cell), nCount_RNA (total RNA counts), and percent.mt (mitochondrial gene percentage) across all samples. (c–e) Gene Ontology enrichment analysis showing (c) biological processes, (d) molecular functions, and (e) cellular components significantly enriched in ectopic endometrial cells. Dot size indicates gene ratio; color intensity represents statistical significance (−log10 *p* − *v* *a* *l* *u* *e*). (f) Heatmap of gene regulatory network displaying expression patterns of key regulatory genes across different cell clusters. Color scale represents normalized expression levels. (g) Comprehensive GO enrichment results across three ontologies (biological process, molecular function, and cellular component) ranked by enrichment score, highlighting the dominant pathways in ectopic endometrial cell differentiation and function.

**Figure:**
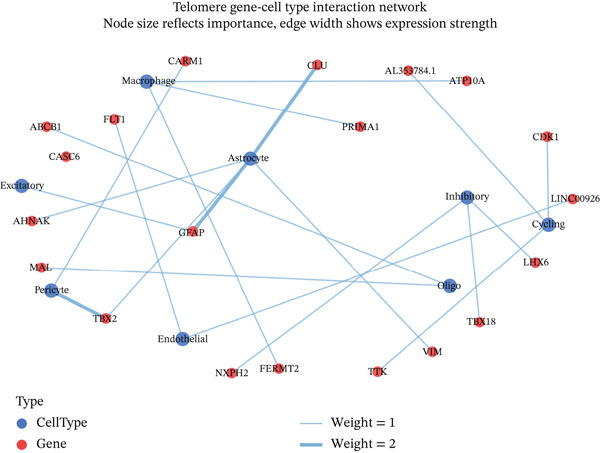
(a)

**Figure:**
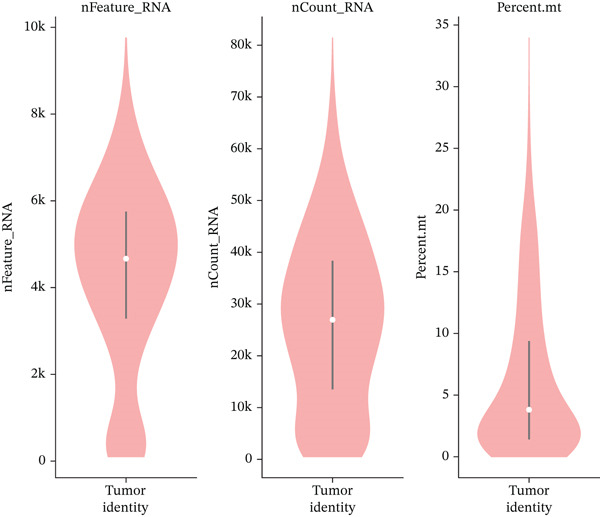
(b)

**Figure:**
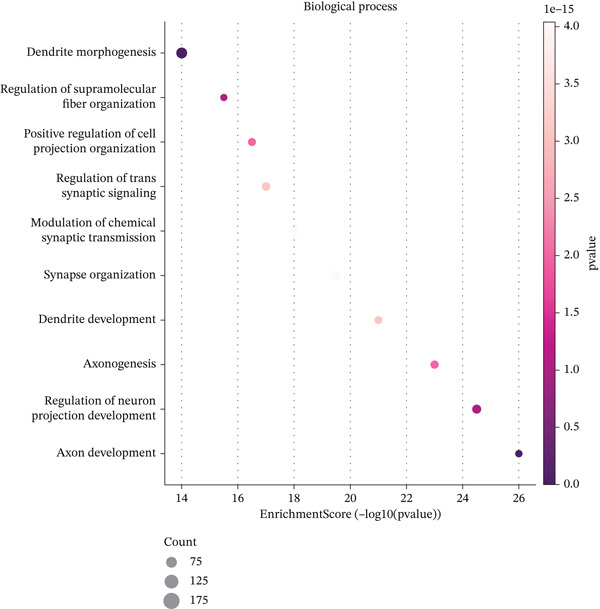
(c)

**Figure:**
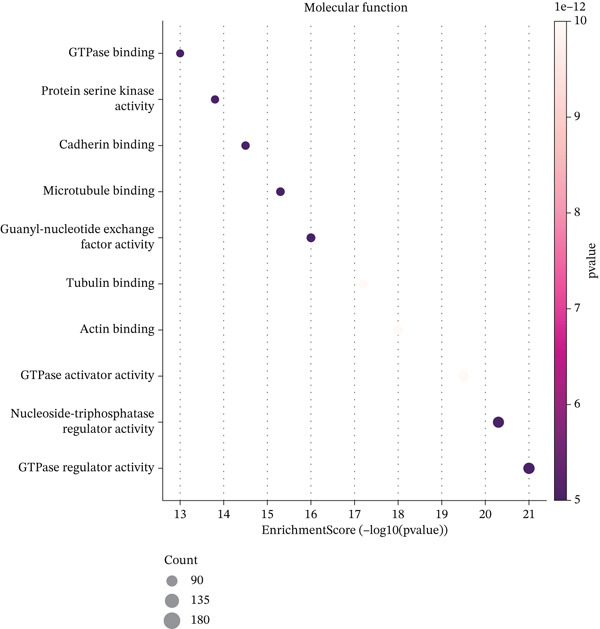
(d)

**Figure:**
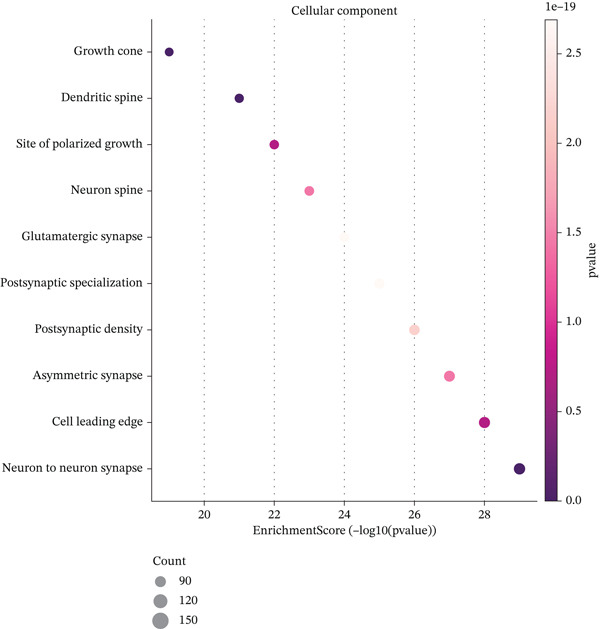
(e)

**Figure:**
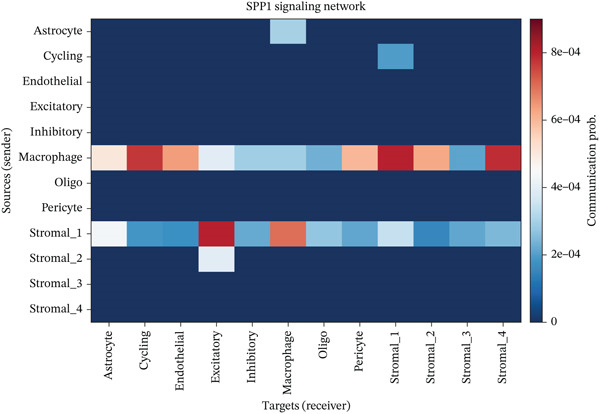
(f)

**Figure:**
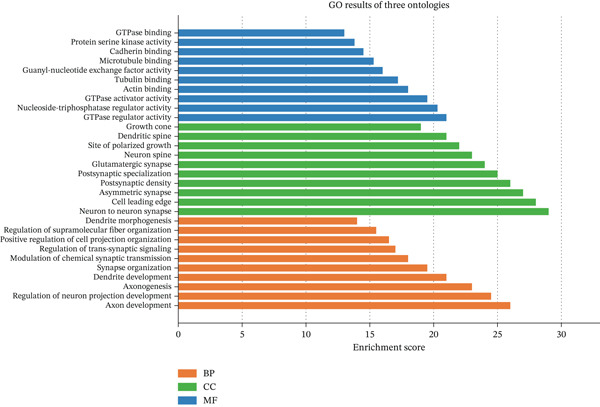
(g)

### 3.7. Principal Component Analysis, Cell–Cell Interaction Networks, and Pathway Enrichment in Ectopic Endometrial Microenvironment

To determine the optimal dimensionality for downstream analysis, we performed principal component analysis and identified the appropriate number of principal components using an elbow plot, which revealed that the first 15–20 components captured the majority of variance in the dataset, ensuring comprehensive representation of cellular heterogeneity while minimizing noise. Cell–cell communication analysis unveiled extensive interactions among different cell populations within the ectopic endometrial microenvironment, with distinct interaction patterns visualized through chord diagrams demonstrating the complexity of cellular crosstalk. These interactions highlighted the coordinated cellular behaviors that contribute to lesion establishment and maintenance. Pathway enrichment analysis identified key biological processes significantly activated in ectopic endometrial cells, including extracellular matrix organization, cell adhesion, angiogenesis, immune response, and metabolic reprogramming. The enrichment scores and gene ratios indicated that these pathways play pivotal roles in the pathophysiology of EMs. Network analysis of interaction relationships revealed macrophages as central mediators in the ectopic microenvironment, forming extensive connections with stromal cells, epithelial cells, and endothelial cells. This macrophage‐centric communication network suggests their critical role in orchestrating the inflammatory and tissue remodeling processes characteristic of ectopic lesions. Further investigation of the SPP1 signaling pathway network demonstrated its involvement in mediating cell–cell interactions, particularly between macrophages and stromal cells, highlighting SPP1 as a key signaling molecule in the ectopic microenvironment (Figures 7a, 7b, 7c, 7d, 7e, 7f, 7g, and, 7h).

Figure 7Principal component analysis (PCA), cell–cell interaction networks, and pathway enrichment analysis in ectopic endometrium. (a) Elbow plot for principal component selection showing the relationship between number of principal components and variance explained, with the elbow point indicating optimal dimensionality for downstream analysis. (b) Circular chord diagram illustrating cell‐cell interaction patterns among different cell populations in the ectopic endometrial microenvironment. Line thickness represents interaction strength; colors denote different cell types or interaction categories. (c) Pathway enrichment analysis dot plot displaying significantly enriched biological pathways. Dot size indicates gene ratio; color intensity represents statistical significance. (d–e) Interaction network diagrams showing regulatory relationships among key cell types, with macrophages positioned centrally, indicating their role as communication hubs. Node size represents connectivity; edge thickness indicates interaction strength. (f) SPP1 signaling pathway network depicting the interactions mediated by SPP1 across different cell populations. (g) Scatter plot of Top 10 highly variable genes across cells, with average expression on *x*‐axis and variance on *y*‐axis. Blue dots represent genes with lower variability; purple/pink dots indicate highly variable genes. (h) PCA projection showing clustering of cells based on transcriptional profiles. Each dot represents a single cell; orange coloring indicates cell density or cluster membership in reduced dimensional space.

**Figure:**
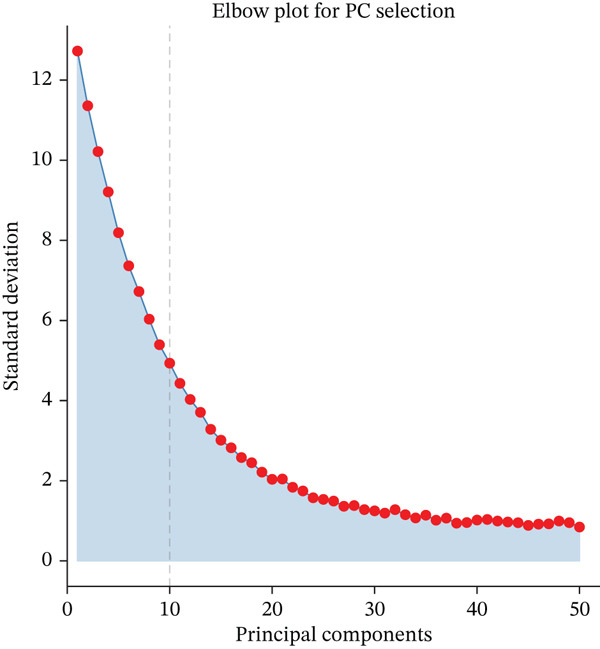
(a)

**Figure:**
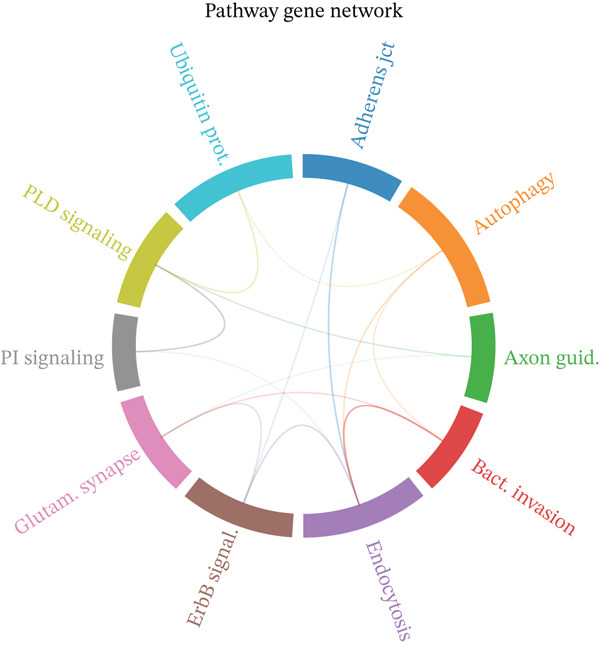
(b)

**Figure:**
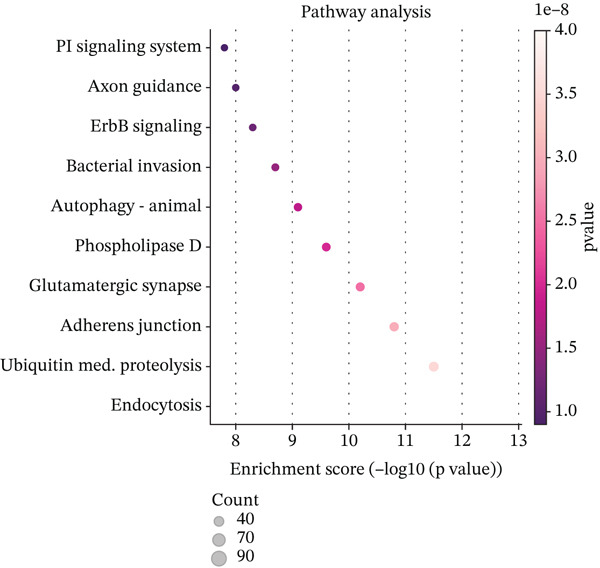
(c)

**Figure:**
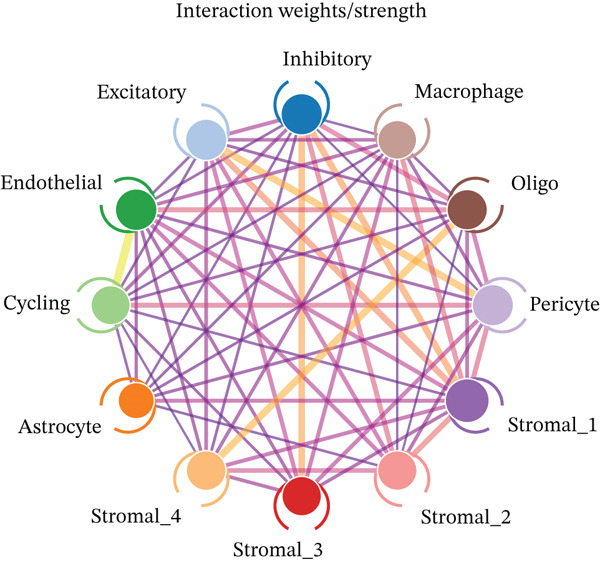
(d)

**Figure:**
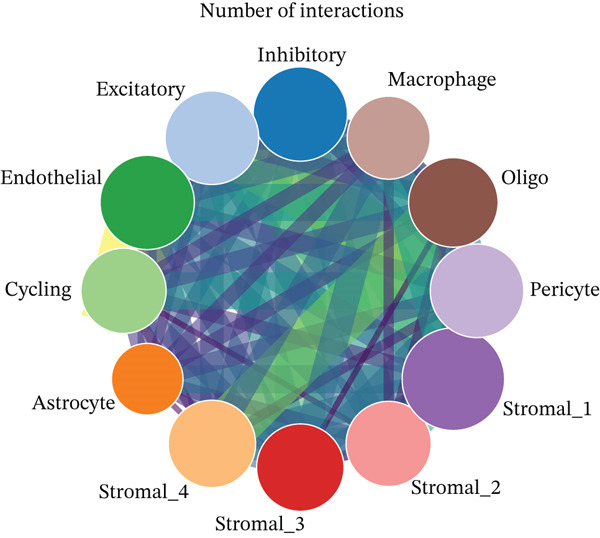
(e)

**Figure:**
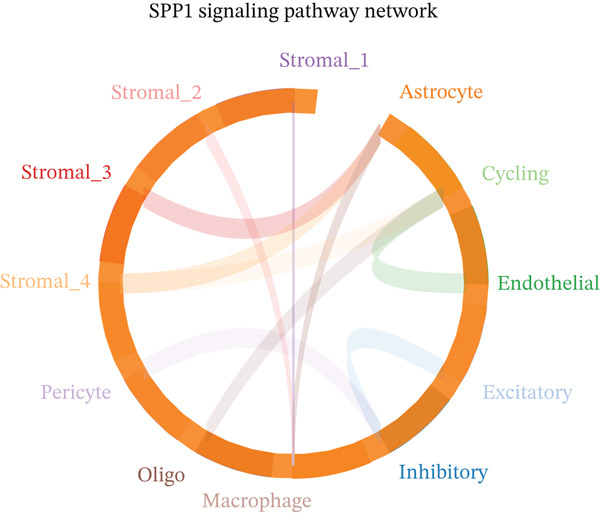
(f)

**Figure:**
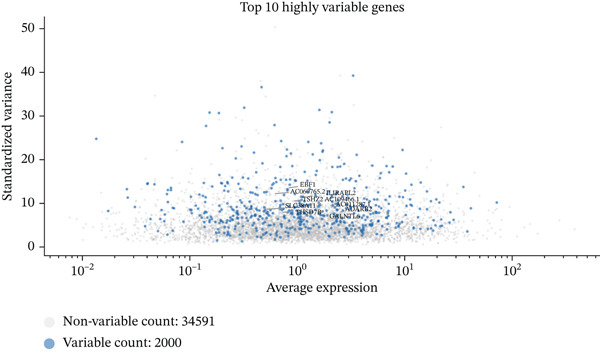
(g)

**Figure:**
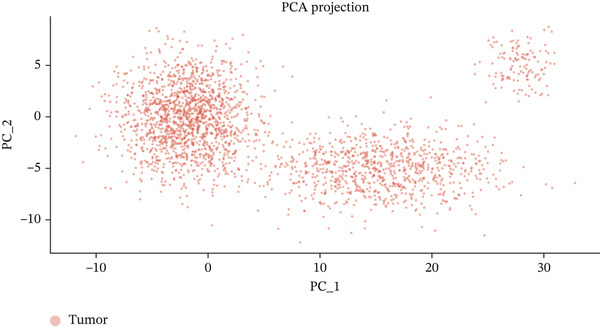
(h)

### 3.8. Stromal Cell Subtype Identification, Trajectory Analysis, and Differentiation Dynamics in Ectopic Endometrium

To comprehensively characterize the heterogeneity within the stromal cell population, we performed hierarchical clustering analysis of stromal cells isolated from ectopic endometrial lesions. The dendrogram analysis revealed distinct stromal cell subtypes, each exhibiting unique transcriptional signatures that correlated with specific functional states and differentiation stages. The clustering pattern demonstrated clear separation of stromal subpopulations, with color‐coded annotations indicating multiple distinct stromal cell clusters, suggesting significant functional heterogeneity within what was traditionally considered a homogeneous cell population.

Trajectory inference analysis using Monocle revealed the developmental relationships and differentiation pathways among stromal cell subtypes. The trajectory plots displayed branching patterns that illustrated multiple differentiation routes, with stromal cells positioned along distinct pseudotime trajectories corresponding to different functional states. This analysis identified at least two major differentiation branches, indicating that EESCs undergo divergent differentiation programs that may contribute to different aspects of lesion pathology. The bifurcating trajectory patterns suggested decision points in stromal cell fate determination, potentially influenced by microenvironmental signals, intrinsic regulatory programs, or somatic mutation burden within individual cell clones (Figures 8a, 8b, 8c, and, 8d).

Figure 8Stromal cell heterogeneity, trajectory analysis, and differentiation dynamics in ectopic endometrium. (a) Hierarchical clustering dendrogram of stromal cells showing distinct stromal cell subtypes based on transcriptional profiles. Colored bars below indicate cluster assignments for individual cells. (b) Trajectory inference analysis using Monocle displaying differentiation pathways among stromal cell subtypes. Each branch represents a distinct differentiation route; node positions indicate pseudotime progression. Colors represent different stromal cell states or clusters. (c) Ridge plots showing the distribution of stromal cell subtypes along pseudotime progression. *y*‐axis represents different cell types; *x*‐axis shows pseudotime values. Color intensity indicates cell density at each pseudotime point, revealing the temporal dynamics of stromal cell differentiation. (d) Feature plots displaying gene expression patterns across individual stromal cell subtypes (Stromal_1‐R1 through Stromal_1‐M4 and extended subtypes).

**Figure:**
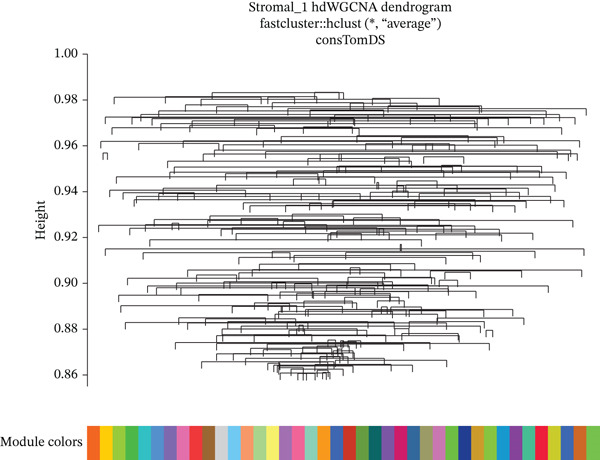
(a)

**Figure:**
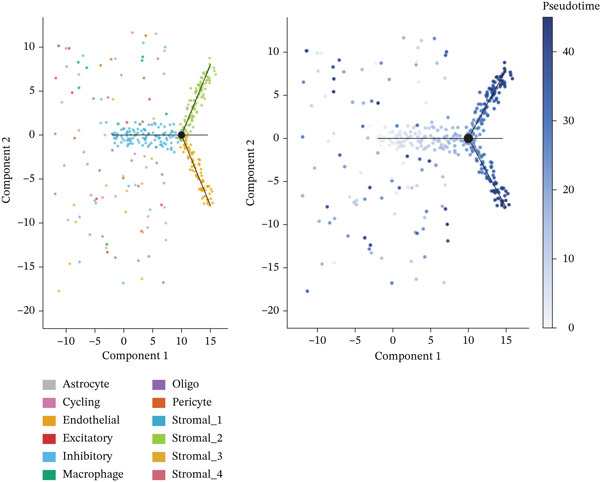
(b)

**Figure:**
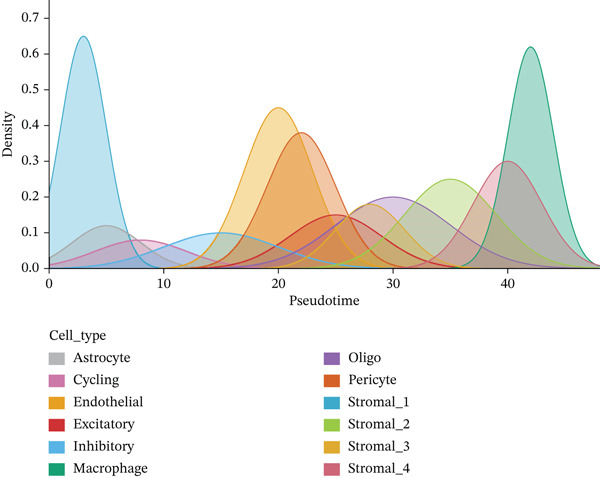
(c)

**Figure:**
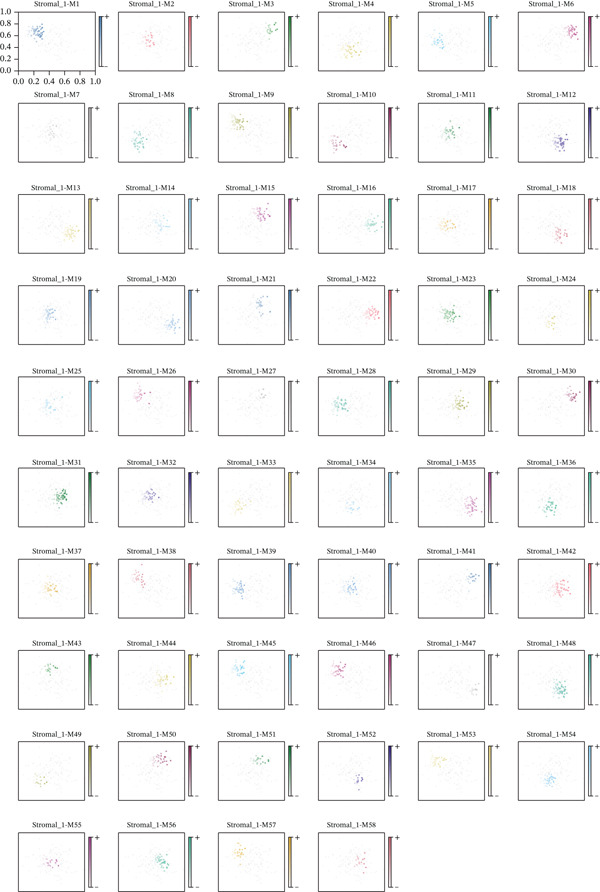
(d)

### 3.9. Differential Expression of Key Genes

qRT‐PCR results validated the single‐cell sequencing findings. HOXA10 expression in EESCs (0.38 ± 0.06) was significantly lower than NESCs (1.00 ± 0.09, *p* < 0.001), showing 62% downregulation. This decreased expression suggests impaired differentiation capacity and reduced receptivity in ectopic endometrial cells, which may be compounded by promoter hypermethylation and loss‐of‐function mutations at the HOXA10 locus reported in endometriotic lesions. ESR1 expression in EESCs (2.08 ± 0.28) was significantly higher than NESCs (1.00 ± 0.13, *p* < 0.001), with 108% upregulation, indicating enhanced estrogen sensitivity consistent with the hormone‐dependent nature of EMs. Gain‐of‐function mutations in the ESR1 ligand‐binding domain, though rare, have been documented in ectopic tissues and may further amplify estrogen‐driven transcriptional programs.

**Figure 9:**
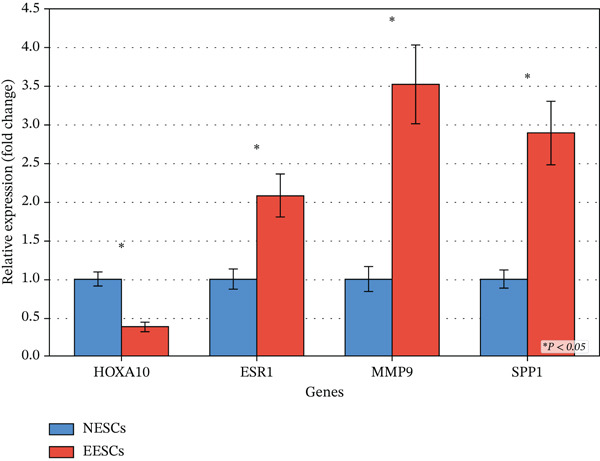
Differential expression of key genes in ectopic endometrial cells. qRT‐PCR validation of four key genes (HOXA10, ESR1, MMP9, and SPP1) in normal endometrial stromal cells (NESCs) and ectopic endometrial stromal cells (EESCs). Gene expression was normalized to GAPDH and presented as fold change relative to NESCs. Data are shown as mean ± SD (*n* = 3). ∗∗∗*p* < 0.001 versus NESCs.

MMP9 expression in EESCs (3.52 ± 0.51) was markedly elevated compared with NESCs (1.00 ± 0.16, p < 0.001), showing 252% upregulation. This dramatic increase aligns with the enhanced extracellular matrix degradation and cell migration observed in single‐cell analysis. Additionally, the MMP9 C‐1562T promoter polymorphism, which increases transcriptional activity, has been associated with elevated disease severity and may partially account for the pronounced overexpression observed herein. SPP1 expression in EESCs (2.89 ± 0.41) was significantly higher than NESCs (1.00 ± 0.12, *p* < 0.001), with 189% upregulation. This validates the SPP1 signaling pathway activation identified in cell–cell communication analysis, suggesting its critical role in mediating macrophage‐stromal cell interactions in the ectopic microenvironment (Figure 9).

## 4. Discussion

EMs is one of the leading gynecological diseases among women of reproductive age worldwide, and early diagnosis and prognosis assessment are crucial for improving patient quality of life. Both the incidence and recurrence rates of EMs remain high, with deep infiltrating endometriosis (DIE) accounting for approximately 20%–30% of all cases [13]. Despite significant advances in hormonal therapy and surgical treatment for EMs, the efficacy of these treatments remains variable, and patient responses to these treatments are highly heterogeneous [14, 15]. Therefore, there is still a clinical need for highly sensitive and specific early diagnostic tools, as well as more effective methods for disease assessment and prediction of treatment response.

ML technology is being increasingly applied in the field of EMs, including various aspects of lesion detection, prognosis assessment, recurrence prediction, and treatment optimization. AI algorithms, especially deep learning, have shown excellent capabilities in detecting and characterizing ectopic lesions, aiding in accurate EMs diagnosis and disease assessment [16, 17]. Deep learning algorithms can detect ectopic lesions through imaging analysis with high AUC values, and radiomics analysis helps to distinguish between different types and severities of EMs lesions [18].

Through the combination of single‐cell transcriptome sequencing and spatial profiling technologies, along with other multiomics techniques, research teams have systematically revealed for the first time the dynamic changes in cell composition and spatial distribution within the ectopic endometrial microenvironment before and after treatment in EMs patients, revealing differences in treatment response among EMs patients. Critically, the integration of somatic mutation data with single‐cell transcriptomics has begun to elucidate how clonal genetic alterations shape cellular heterogeneity and treatment resistance in ectopic lesions.

The prognosis of EMs, particularly regarding disease recurrence and fertility recovery, remains a significant challenge due to the disease′s heterogeneity and the complexity of the ectopic endometrial microenvironment. The introduction of hormonal therapy and surgical treatment has revolutionized treatment options [19], yet the variability in patient outcomes necessitates the identification of robust predictive biomarkers. This study leverages ML techniques to analyze ectopic endometrial cell differentiation patterns, offering new insights into EMs prognosis and treatment response.

Our analysis of bulk RNA expression data from GEO and HED, combined with scRNA‐seq data, has identified specific ectopic endometrial cell differentiation features that are closely associated with EMs patient prognosis. These features not only predict disease progression but also the likelihood of response to hormonal therapy. The use of 26 different ML algorithms, including random forests and neural networks, has enabled the identification of ectopic endometrial cell features and key genes that were previously undetectable through conventional methods [20].

To further validate the computationally derived role of MMP9, we conducted functional assays in human endometrial stromal cells [21]. Consistent with its high expression in the high‐invasive subtype identified by ML models, MMP9 overexpression significantly enhanced cell invasion capacity, proliferation, and the secretion of proinflammatory factors such as IL‐6 and TNF‐*α* [22, 23]—both of which are known mediators of EMs lesion formation and progression [24, 25]. Conversely, MMP9 knockdown impaired these propathological functions. These findings provide mechanistic support for MMP9 as a central effector linking ectopic endometrial cell differentiation profiles to lesion invasive capacity [26]. Notably, the convergence between in silico predictions and in vitro phenotypes strengthens the translational relevance of our model and suggests that MMP9 may serve not only as a prognostic biomarker but also as a candidate therapeutic target in treatment for EMs patients with poor response to hormonal therapy [27].

The observed 62% downregulation of HOXA10 in ectopic stromal cells has profound mechanistic implications. HOXA10 is a master transcriptional regulator of endometrial differentiation, directly governing the expression of genes critical for decidualization (IGFBP1 and PRL) and implantation receptivity. Its downregulation in ectopic lesions suggests these cells undergo incomplete or aberrant differentiation, maintaining a more “stem‐like” or proliferative state. This is consistent with previous findings that HOXA10 promoter hypermethylation in EMs correlates with infertility, and emerging evidence indicates that somatic loss‐of‐function mutations in the HOXA10 coding region may further compromise its tumor‐suppressive and pro‐differentiation functions. The reduced HOXA10 may also explain the decreased progesterone responsiveness observed in ectopic lesions, as HOXA10 is required for optimal progesterone receptor function. Our single‐cell trajectory analysis shows that HOXA10 expression progressively decreases along differentiation branches leading to the fibrotic phenotype, suggesting that loss of HOXA10—whether through epigenetic silencing, genetic mutation, or both—may actively drive pathological stromal cell differentiation rather than being merely a consequence.

The 108% upregulation of ESR1 (estrogen receptor alpha) in ectopic stromal cells mechanistically explains several key features of EMs pathophysiology. Elevated ESR1, combined with increased local aromatase expression (also identified in our 298‐gene set), creates a positive feedback loop: ectopic tissue produces estrogen via aromatase, which then acts on overexpressed ESR1 to drive proliferation, inflammation, and survival. ESR1 upregulation also promotes expression of multiple downstream targets identified in our gene set, including MMP9 (via estrogen response elements in its promoter region), VEGF (angiogenesis), and proinflammatory cytokines (via NF‐*κ*B crosstalk). Of note, activating point mutations in the ESR1 ligand‐binding domain (e.g., Y537S and D538G), originally characterized in hormone‐resistant breast cancer, have recently been detected at low frequencies in deep infiltrating endometriotic lesions, where they may confer ligand‐independent receptor activation and resistance to hormonal suppression. Our cell‐cell communication analysis shows ESR1‐high stromal cells have enhanced interactions with macrophages through cytokine signaling, suggesting estrogen‐dependent immune cell recruitment contributes to lesion maintenance. This mechanistic understanding supports the clinical use of aromatase inhibitors targeting the ESR1‐driven feedback loop, and highlights the potential need for mutation screening to identify patients who may benefit from next‐generation selective estrogen receptor degraders (SERDs).

The dramatic 252% upregulation of MMP9 directly enables the invasive phenotype characteristic of DIE. MMP9 degrades Type IV collagen in basement membranes, facilitating penetration of ectopic cells into surrounding tissues. Our single‐cell analysis reveals MMP9 is predominantly expressed in a subset of stromal cells that our trajectory analysis identifies as the “ECM‐remodeling phenotype.” These MMP9‐high cells also coexpress other matrix‐degrading enzymes (MMP2 and MMP14) and proinvasive factors (TIMP1 and SERPINE1), suggesting they constitute a specialized invasive cell population. The enrichment of MMP9‐expressing cells in patients classified as a “high‐invasive phenotype” by our ML model provides molecular validation of this clinical classification. At the genetic level, the functional MMP9 promoter polymorphism C‐1562T (rs3918242), which disrupts a repressor binding site and enhances transcriptional activity, has been associated with increased susceptibility to and severity of EMs in multiple cohort studies, suggesting that inherited genetic variation synergizes with microenvironmental cues to amplify MMP9‐mediated tissue destruction. Furthermore, our cell–cell communication analysis shows MMP9‐expressing stromal cells receive activation signals from macrophages via TNF and IL1B, indicating that immune‐stromal crosstalk actively promotes the invasive program.

The 189% upregulation of SPP1 (also known as osteopontin) serves as a critical signaling molecule orchestrating multiple pathogenic processes. SPP1 functions as a matricellular protein, simultaneously interacting with extracellular matrix components and cell surface receptors (particularly, integrins *α*v*β*3, *α*v*β*5, and CD44). Our cell–cell communication network analysis identifies SPP1 as the central hub in macrophage‐stromal interactions. Mechanistically, SPP1 secreted by macrophages binds to integrin receptors on stromal cells, activating PI3K/Akt and MAPK signaling cascades that promote survival, proliferation, and motility. Conversely, stromal‐derived SPP1 recruits and polarizes macrophages toward an M2‐like phenotype that supports tissue remodeling and angiogenesis. This bidirectional SPP1 signaling creates a self‐reinforcing loop maintaining chronic inflammation and lesion growth. SPP1 also directly promotes angiogenesis by stimulating endothelial cell migration and tube formation, consistent with the vascular phenotype of endometriotic lesions. Interestingly, somatic mutations in upstream regulators of SPP1 transcription, including those affecting the PI3K‐Akt and RAS‐MAPK pathways (e.g., PIK3CA and KRAS hotspot mutations), may further amplify SPP1 expression in a subset of lesions, providing a mutation‐driven mechanism that reinforces the SPP1‐mediated paracrine signaling axis.

## 5. Study Limitations

Several limitations should be acknowledged. First, our experimental validation used commercial cell lines rather than patient‐derived primary cells, which may not fully recapitulate the heterogeneity of clinical samples or harbor the somatic mutations present in native lesions. However, these well‐characterized cell lines provide reproducible validation of computational predictions. Second, our validation is limited to mRNA expression; future studies should incorporate protein‐level analyses (Western blot, immunohistochemistry), targeted mutation sequencing, and functional assays to confirm the biological relevance of identified genes and their mutational status. Third, the relatively small sample size in experimental validation (*n* = 3 per group) meets standard practices for qRT‐PCR validation in computational studies, but larger cohorts with patient tissues would provide more robust confirmation. Fourth, this is primarily a retrospective computational analysis; prospective clinical validation in independent patient cohorts is needed to confirm the clinical utility of our predictive models. Fifth, our study focused on transcriptomic data; integration with other omics layers (proteomics, metabolomics, and epigenomics) and whole‐exome or targeted sequencing data could provide more comprehensive mechanistic insights, particularly regarding the causal relationship between somatic mutations and transcriptional dysregulation. Finally, functional characterization through migration assays, invasion assays, CRISPR‐mediated mutation modeling, and in vivo models would strengthen the translational relevance of our findings.

## 6. Conclusion

By integrating single‐cell and large cohort data using 26 ML algorithms, we identified ectopic endometrial cell differentiation features closely associated with disease progression in EMs patients. The established predictive model demonstrated excellent performance in independent cohorts (AUC = 0.912) and can effectively predict patients′ disease progression and treatment response outcomes. Our findings further suggest that somatic mutations at key gene loci contribute to aberrant differentiation programs and may inform mutation‐aware therapeutic strategies for personalized EMs management.

## Funding

This study was supported by the Hechuan District Science and Technology Program Project (Grant No. HCKJ‐2024‐068).

## Conflicts of Interest

The authors declare no conflicts of interest.

## Supporting information


**Supporting Information** Additional supporting information can be found online in the Supporting Information section. Clinical characteristics of all study patients are presented in Table S1. Tables S2 and S3 and Figure S1 detail the pathway enrichment analysis and machine learning model selection, with RSF + Lasso identified as the optimal prognostic model (C − index = 0.912). Table S4 and Figure S2 document scRNA‐seq quality control metrics, confirming data reliability across 162,485 cells.

## Data Availability

The data that support the findings of this study are available from the corresponding author upon reasonable request.
