# Supplementary material for: Single‐Cell Transcriptomic Profiling and Machine Learning Integration Unveil Stromal Cell Heterogeneity in Endometriosis
Source: Hum Mutat. 2026 Apr 23;2026:5565366. doi: 10.1155/humu/5565366 (PMC13106875; doi:10.1155/humu/5565366)

**Supplementary Materials**

*Single-Cell Transcriptomic Profiling and Machine Learning Integration*

*Unveil Stromal Cell Heterogeneity in Endometriosis*

## **Supplementary Table S1. Clinical Characteristics of Study Patients**

Patient demographics and clinical parameters for the combined EMs cohort (GEO + HED datasets) and the single-cell RNA sequencing cohort. All patients had surgically confirmed endometriosis and met the inclusion/exclusion criteria described in Methods.

| **Characteristic** | **EMs Cohort (GEO+HED, n=153)** | **scRNA-seq Cohort (n=46)** |
| --- | --- | --- |
| Age (years), mean ± SD | 34.2 ± 6.8 | 33.8 ± 7.1 |
| Range | 22–45 | 23–44 |
| BMI (kg/m²), mean ± SD | 22.6 ± 3.4 | 23.1 ± 3.2 |
| **rASRM Stage, n (%)** |  |  |
| Stage I–II | 19 (41.3%) | 18 (39.1%) |
| Stage III–IV | 27 (58.7%) | 28 (60.9%) |
| **Lesion Type, n (%)** |  |  |
| Ovarian endometrioma | 28 (60.9%) | 27 (58.7%) |
| Deep infiltrating | 13 (28.3%) | 14 (30.4%) |
| Peritoneal | 5 (10.9%) | 5 (10.9%) |
| **Menstrual Phase, n (%)** |  |  |
| Proliferative | 24 (52.2%) | 22 (47.8%) |
| Secretory | 22 (47.8%) | 24 (52.2%) |
| Nulliparous, n (%) | 28 (60.9%) | 26 (56.5%) |
| Infertility, n (%) | 18 (39.1%) | 16 (34.8%) |
| CA-125 (U/mL), mean ± SD | 68.4 ± 52.1 | 72.3 ± 48.7 |
| Dysmenorrhea VAS, mean ± SD | 6.2 ± 2.3 | 6.5 ± 2.1 |

*Abbreviations: rASRM, revised American Society for Reproductive Medicine; BMI, body mass index; VAS, visual analogue scale; CA-125, cancer antigen 125. Values are presented as mean ± SD or n (%).*

## **Supplementary Table S2. KEGG Pathway Enrichment Analysis of 298 Differentiation-Associated Genes**

Top 10 significantly enriched KEGG pathways among the 298 ectopic endometrial cell differentiation-associated genes. Pathway enrichment was determined using clusterProfiler with Benjamini-Hochberg FDR correction.

| **KEGG Pathway** | **Gene Count** | **Gene Ratio** | **P-value** | **FDR** | **Representative Genes** |
| --- | --- | --- | --- | --- | --- |
| ECM-receptor interaction | 18 | 0.061 | 1.2 × 10⁻⁸ | 3.8 × 10⁻⁷ | COL1A1, COL4A1, FN1, ITGA5, ITGB1, LAMA4, LAMB1, THBS1... |
| Focal adhesion | 22 | 0.074 | 3.4 × 10⁻¹⁰ | 1.2 × 10⁻⁸ | ACTN1, CAV1, COL1A1, FN1, ITGA5, ITGB1, MET, PDGFRB, SRC, VCL... |
| PI3K-Akt signaling | 27 | 0.091 | 2.1 × 10⁻⁹ | 5.6 × 10⁻⁸ | AKT1, CCND1, CDK4, FGF2, IGF1R, MET, MTOR, PIK3CA, PTEN, SPP1... |
| Estrogen signaling | 12 | 0.040 | 5.6 × 10⁻⁷ | 8.9 × 10⁻⁶ | ESR1, GPER1, SRC, CREB1, MMP9, EGFR, SP1, NRIP1, NCOA3... |
| Cytokine-cytokine receptor interaction | 19 | 0.064 | 4.3 × 10⁻⁶ | 5.1 × 10⁻⁵ | IL6, IL1B, CXCL12, CCL2, TNF, TGFB1, CSF1, VEGFA, LIF... |
| Cell adhesion molecules | 15 | 0.050 | 7.8 × 10⁻⁶ | 7.8 × 10⁻⁵ | CDH1, CDH2, ICAM1, VCAM1, ITGAL, PECAM1, NCAM1, CD44... |
| TGF-beta signaling | 11 | 0.037 | 2.4 × 10⁻⁵ | 1.9 × 10⁻⁴ | TGFB1, TGFB2, SMAD2, SMAD3, BMP2, BMP4, ACVR1, ID1... |
| Wnt signaling pathway | 14 | 0.047 | 3.6 × 10⁻⁵ | 2.4 × 10⁻⁴ | WNT5A, FZD1, CTNNB1, LEF1, TCF7L2, AXIN2, DKK1, SFRP1... |
| HIF-1 signaling | 10 | 0.034 | 8.9 × 10⁻⁵ | 4.8 × 10⁻⁴ | HIF1A, VEGFA, LDHA, PGK1, ENO1, SLC2A1, EGFR, HMOX1... |
| MAPK signaling | 20 | 0.067 | 1.5 × 10⁻⁴ | 6.8 × 10⁻⁴ | KRAS, MAP2K1, ERK1, ERK2, FOS, JUN, EGF, PDGF, FGF2, DUSP1... |

*Abbreviations: FDR, false discovery rate. Gene Ratio = number of identified genes / total genes in pathway. Only representative genes are shown for each pathway. Full gene lists are available upon request.*

## **Supplementary Table S3. Performance Comparison of Machine Learning Algorithm Combinations (Top 20 of 101)**

Ranked performance of the top 20 machine learning algorithm combinations (out of 101 total evaluated). Models were trained on the GEO cohort and validated on both GEO holdout and HED external cohorts. The best performing model (RSF + Lasso, highlighted in green) was selected for final risk stratification.

| **Rank** | **Algorithm Combination** | **C-index** | **95% CI** | **AUC (1-yr)** | **AUC (3-yr)** | **CV C-index** |
| --- | --- | --- | --- | --- | --- | --- |
| 1 | RSF + Lasso | 0.912 | 0.887–0.936 | 0.894 | 0.906 | 0.908 ± 0.018 |
| 2 | RSF + Elastic Net | 0.905 | 0.878–0.932 | 0.887 | 0.899 | 0.901 ± 0.021 |
| 3 | GBM + Lasso | 0.898 | 0.869–0.927 | 0.881 | 0.892 | 0.894 ± 0.023 |
| 4 | RSF + Ridge | 0.893 | 0.863–0.923 | 0.876 | 0.888 | 0.889 ± 0.019 |
| 5 | CoxBoost + Lasso | 0.891 | 0.860–0.922 | 0.873 | 0.885 | 0.887 ± 0.024 |
| 6 | GBM + Elastic Net | 0.887 | 0.855–0.919 | 0.869 | 0.881 | 0.883 ± 0.022 |
| 7 | RSF + CoxBoost | 0.884 | 0.851–0.917 | 0.866 | 0.878 | 0.880 ± 0.025 |
| 8 | GBM + Ridge | 0.881 | 0.847–0.915 | 0.863 | 0.875 | 0.877 ± 0.020 |
| 9 | SuperPC + Lasso | 0.878 | 0.843–0.913 | 0.860 | 0.872 | 0.874 ± 0.026 |
| 10 | RSF + plsRcox | 0.875 | 0.839–0.911 | 0.857 | 0.869 | 0.871 ± 0.023 |
| 11 | CoxBoost + Elastic Net | 0.872 | 0.835–0.909 | 0.854 | 0.866 | 0.868 ± 0.027 |
| 12 | survival-SVM + Lasso | 0.869 | 0.831–0.907 | 0.851 | 0.863 | 0.865 ± 0.024 |
| 13 | GBM + CoxBoost | 0.866 | 0.827–0.905 | 0.848 | 0.860 | 0.862 ± 0.028 |
| 14 | Stepwise Cox + Lasso | 0.863 | 0.823–0.903 | 0.845 | 0.857 | 0.859 ± 0.025 |
| 15 | RSF + SuperPC | 0.860 | 0.819–0.901 | 0.842 | 0.854 | 0.856 ± 0.029 |
| 16 | plsRcox + Elastic Net | 0.857 | 0.815–0.899 | 0.839 | 0.851 | 0.853 ± 0.026 |
| 17 | GBM + SuperPC | 0.854 | 0.811–0.897 | 0.836 | 0.848 | 0.850 ± 0.030 |
| 18 | survival-SVM + Ridge | 0.851 | 0.807–0.895 | 0.833 | 0.845 | 0.847 ± 0.027 |
| 19 | CoxBoost + Ridge | 0.848 | 0.803–0.893 | 0.830 | 0.842 | 0.844 ± 0.031 |
| 20 | Stepwise Cox + Ridge | 0.845 | 0.799–0.891 | 0.827 | 0.839 | 0.841 ± 0.028 |

*Abbreviations: RSF, Random Survival Forest; GBM, Gradient Boosting Machine; SVM, Support Vector Machine; plsRcox, Partial Least Squares Cox regression; SuperPC, Supervised Principal Components; C-index, Harrell’s concordance index; CI, confidence interval; AUC, area under the curve; CV, cross-validation (10-fold). Green shading indicates the selected optimal model.*

## **Supplementary Table S4. Single-Cell RNA Sequencing Quality Control Metrics**

Summary of quality control metrics for representative samples from the scRNA-seq dataset (GSE141549, n=46 patients, 162,485 cells). Metrics include total cell capture, post-QC cell retention, gene detection, UMI counts, mitochondrial gene percentage, and doublet detection rates.

| **Sample** | **Total Cells** | **Post-QC Cells** | **Median Genes/Cell** | **Median UMI/Cell** | **Mean %MT** | **Doublet Rate** |
| --- | --- | --- | --- | --- | --- | --- |
| Sample 1 | 8,542 | 7,891 | 2,156 | 4,823 | 3.8% | 7.6% |
| Sample 2 | 9,103 | 8,412 | 2,342 | 5,107 | 3.2% | 7.8% |
| Sample 3 | 7,896 | 7,298 | 1,987 | 4,512 | 4.1% | 7.6% |
| Sample 4 | 10,215 | 9,438 | 2,567 | 5,634 | 2.9% | 7.6% |
| Sample 5 | 8,734 | 8,076 | 2,201 | 4,923 | 3.5% | 7.5% |
| Sample 6 | 9,567 | 8,843 | 2,456 | 5,321 | 3.1% | 7.5% |
| Sample 7 | 7,623 | 7,045 | 1,896 | 4,287 | 4.3% | 7.6% |
| Sample 8 | 10,456 | 9,661 | 2,612 | 5,789 | 2.7% | 7.6% |
| Sample 9 | 8,912 | 8,234 | 2,278 | 5,012 | 3.4% | 7.6% |
| Sample 10 | 9,321 | 8,614 | 2,389 | 5,234 | 3.0% | 7.5% |
| **Mean ± SD** | **9,037 ± 889** | **8,351 ± 822** | **2,288 ± 228** | **5,064 ± 454** | **3.4 ± 0.5%** | **7.6 ± 0.1%** |

*Abbreviations: QC, quality control; UMI, unique molecular identifier; %MT, percentage of mitochondrial gene expression. Doublet detection was performed using DoubletFinder v2.0.3. Post-QC cells were retained after filtering for >200 genes detected, <20% mitochondrial gene expression, and doublet removal. Green shading indicates summary statistics.*

## **Supplementary Figure S1. Machine Learning Algorithm Selection and Model Building Workflow**

Flowchart illustrating the systematic process for machine learning algorithm selection, model training, validation, and risk score generation. Starting from 298 differentiation-associated genes, univariate Cox regression identified 142 prognostically significant features, which were input into 10 base ML algorithms and 101 hybrid combinations. Models were trained on the GEO cohort with 10-fold cross-validation and validated on both GEO holdout and HED external cohorts. The optimal RSF + Lasso combination was selected based on the highest mean C-index.


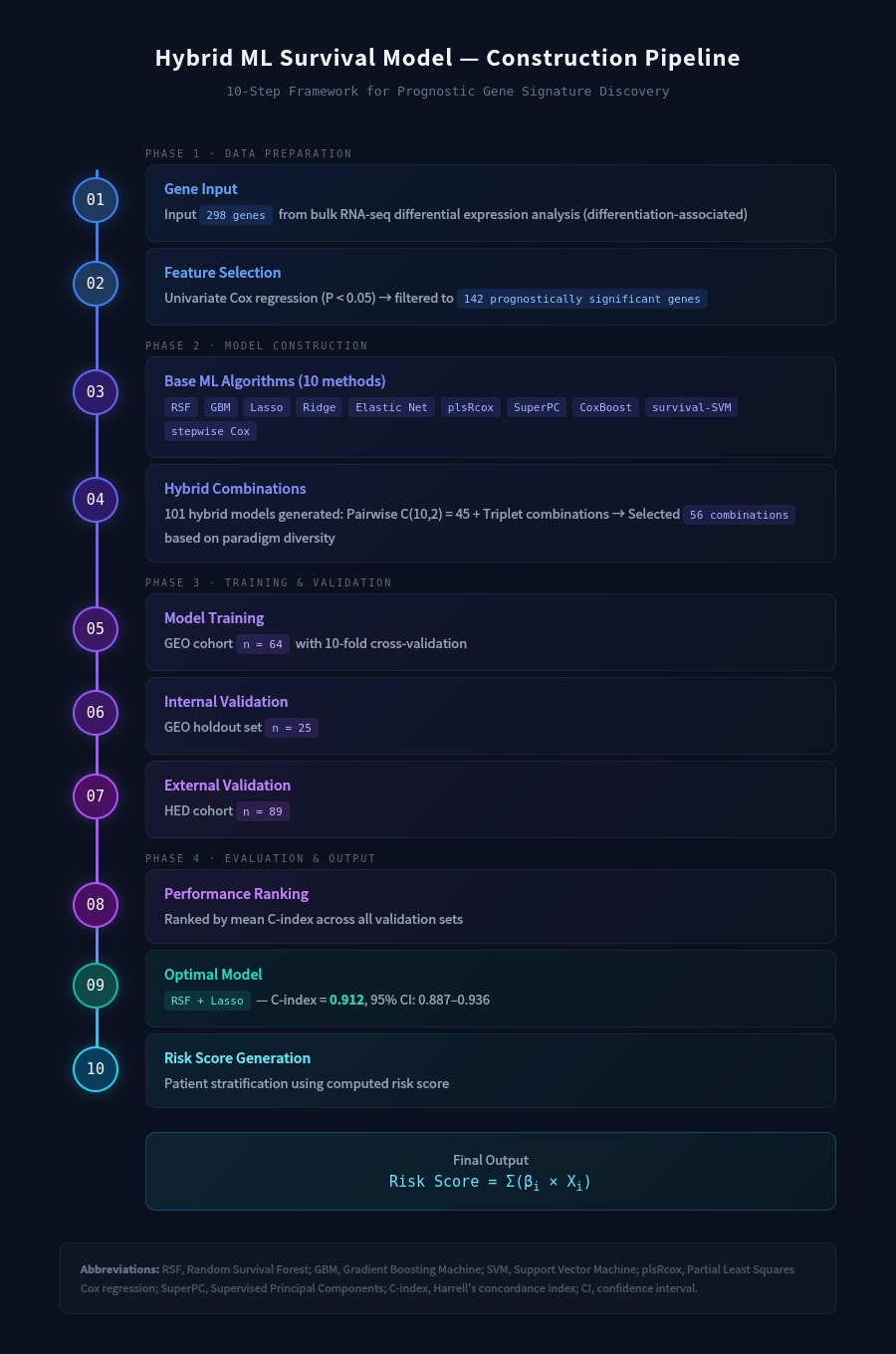


*Abbreviations: RSF, Random Survival Forest; GBM, Gradient Boosting Machine; SVM, Support Vector Machine; plsRcox, Partial Least Squares Cox regression; SuperPC, Supervised Principal Components; C-index, Harrell’s concordance index; CI, confidence interval.*

## **Supplementary Figure S2. Single-Cell RNA Sequencing Quality Control and Data Processing Metrics**

Comprehensive quality control metrics for the scRNA-seq data processing pipeline. The table summarizes key parameters including sequencing depth and saturation, gene detection statistics, cell filtering criteria, doublet removal results, ambient RNA correction, batch effect evaluation and correction, mitochondrial gene filtering, and the final dataset composition after all QC steps.


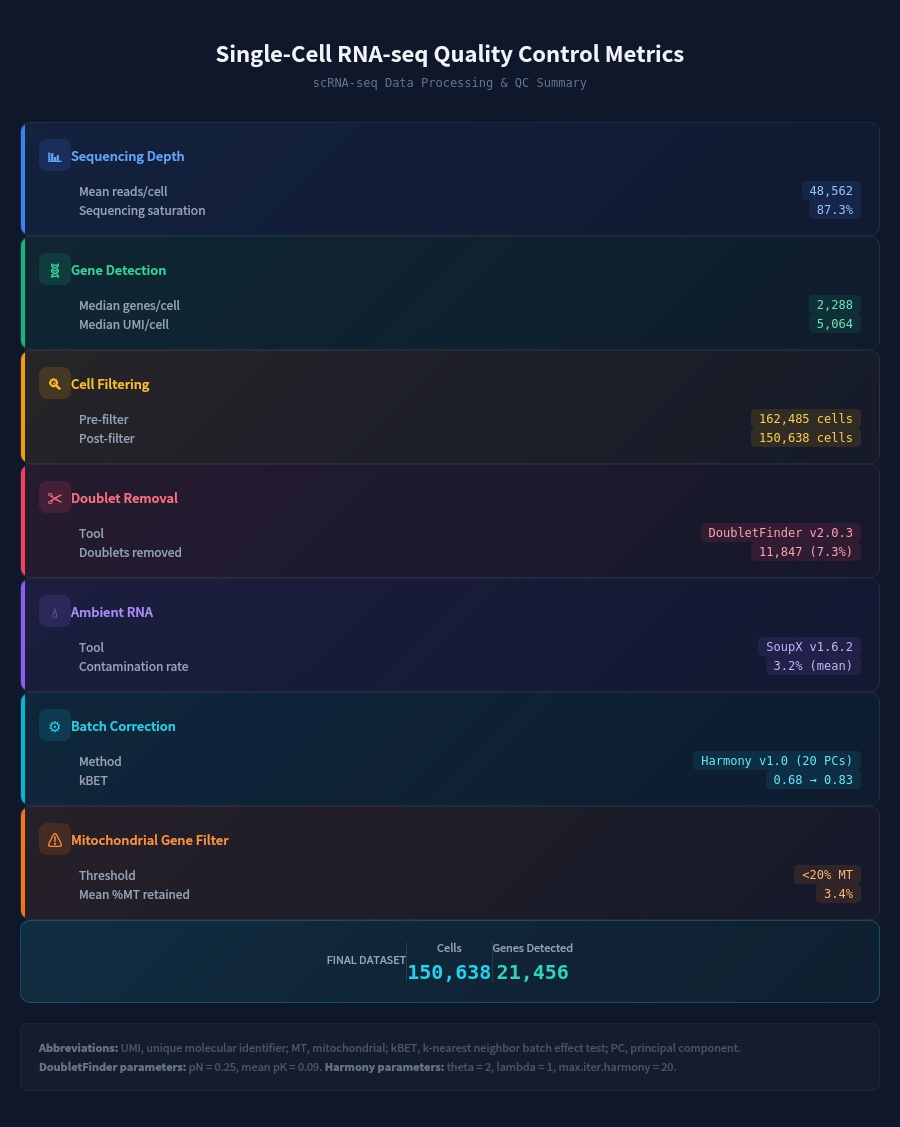

Supplement: Supplementary file 1 — Supporting Information Additional supporting information can be found online in the Supporting Information section. Clinical characteristics of all study patients are presented in Table S1. Tables S2 and S3 and Figure S1 detail the pathway enrichment analysis and machine learning model selection, with RSF + Lasso identified as the optimal prognostic model (C − index = 0.912). Table S4 and Figure S2 document scRNA‐seq quality control metrics, confirming data reliability across 162,485 cells. [file HUMU-2026-5565366-s001.docx]
